# Emotion Regulation Differences Between Gender and Sexuality Groups: A Systematic Review and Meta-Analysis

**DOI:** 10.1007/s10508-025-03276-2

**Published:** 2025-12-16

**Authors:** Jake Camp, Emma Blundell, Patrick Smith, Katharine A. Rimes

**Affiliations:** 1 Department of Psychology, Institute of Psychiatry, Psychology and Neuroscience, King’s College London, London, UK; 2 National and Specialist CAMHS, DBT Service, South London and Maudsley NHS Foundation Trust, Michael Rutter Centre, Maudsley Hospital, London, SE5 8AZ UK

**Keywords:** Emotion regulation, LGBTQ+, Sexual minority, Gender diverse, Meta-analysis

## Abstract

Gender and sexual minority (GSM) people are at increased risk of mental health difficulties compared to their cisgender-heterosexual peers. This is likely because of exposure to minority stressors. Emotion regulation difficulties have been proposed as an important mechanism for mental health disparities in GSM populations. This systematic review and meta-analysis examined whether GSM groups differ from cisgender and heterosexual groups in global and specific aspects of emotion regulation (e.g., emotion awareness, strategies, impulse control, cognitive appraisal, and emotion suppression), and whether emotion dysregulation mediated the relationship between GSM identity and mental health outcomes. We identified 40 eligible studies, of which 28 provided sufficient data for inclusion in the meta-analysis. Results indicated that GSM groups reported higher global emotion regulation difficulties and higher difficulties across most specific domains, compared to cisgender and heterosexual groups. Plurisexual groups (e.g., bisexual+) had higher emotion regulation difficulties compared to monosexual minority groups (e.g., gay/lesbian). A small number of studies also suggested that emotion regulation mediated the relationship between GSM identity and self-harm, suicidality, and eating disorder symptoms. These findings suggest that emotion regulation is a relevant psychological mechanism contributing to mental health disparities in GSM populations. Emotion dysregulation may therefore represent a useful intervention target for reducing health inequalities, alongside broader efforts to address structural and interpersonal minority stress.

**Supplementary Information:**

The online version contains supplementary material available at 10.1007/s10508-025-03276-2.

## Introduction

### Mental Health Disparities in Gender and Sexual Minority Populations

Gender and sexual minority (GSM) populations—such as those who identify as lesbian, gay, bi+/bisexual, trans+/transgender+, queer, pansexual, and with other minority sexual and gender identities—are at increased risk of mental health difficulties compared to their cisgender and heterosexual peers (King et al., 2008; Ross et al., 2018a, 2018b; Semlyen et al., 2016; Williams et al., 2021; Wilson et al., 2023; Wittgens et al., 2022). GSM groups are also at increased risk of suicidal and non-suicidal self-injurious behaviors (Liu et al., 2019; Surace et al., 2021).

These health disparities are considered to arise from exposure to stressors unique to their stigmatized identities, alongside everyday stressors (Dürrbaum & Sattler, 2020; Frost, 2011; Hendricks & Testa, 2012; Meyer, 2003; Pachankis & Clark, 2025; Pitoňák, 2017; Williams & Serpas, 2021). The impact of stigma may be particularly pronounced when the stigma cue is appraised as a threat to the person’s social status and to exceed their ability to cope (Link & Phelan, 2001; Major & O’Brien, 2005). Similarly, the Psychological Mediation Framework (Hatzenbuehler, 2009) proposed that the impact of minority stress on mental health in GSM groups is often mediated by general processes, including emotion regulation, that are not specific to GSM populations.

### The Role of Emotion Regulation

Emotion regulation has been proposed as a key transdiagnostic process linking adversity to psychological distress (Hatzenbuehler et al., 2008, 2009a, 2009b; Hu et al., 2014; Singh et al., 2023). Emotion regulation broadly describes the process of a person modulating the type, duration, and intensity of emotional arousal (Gratz & Roemer, 2004; Gross, 1999). Difficulties with emotion regulation are associated with a number of mental health difficulties (Bud et al., 2023; Hu et al., 2014; Joormann & Stanton, 2016; Kraiss et al., 2020; Leppanen et al., 2022; Visted et al., 2018) and suicidal and non-suicidal self-injurious behaviors (Brereton & McGlinchey, 2020; Colmenero-Navarrete et al., 2022; Rogante et al., 2024; Wolff et al., 2019).

There is also evidence that emotion regulation moderates and mediates the relationship between various vulnerability factors—such as childhood adversity, social isolation, and minority stress—and the development of psychological difficulties and self-injurious behaviors (Chang et al., 2020; English et al., 2018; Inwood & Ferrari, 2018; Hatzenbuehler, 2009; Hatzenbuehler et al., 2008; Kaniuka et al., 2021; Kapatais et al., 2023; Mata-Greve et al., 2022; McCabe et al., 2021; Miu et al., 2022; Poon et al., 2023; Rogante et al., 2024).Therefore, emotion regulation is often regarded as a key psychological intervention target (Cludius et al., 2020; Gratz et al., 2015, 2018; Gross, 2015a; Linehan, 1993).

### Conceptualizing Emotion Regulation

Emotion regulation is widely understood as multidimensional and heterogeneous, with two dominant theoretical frameworks attempting to define and measure this construct (Bridges et al., 2004; Gratz et al., 2015; Sloan et al., 2017).

#### The Process Model of Emotion Regulation

The process model (Gross, 1998, 1999, 2015a, 2015b; Gross & Jazaieri, 2014) defines emotion regulation as a set of strategies to modify affective experiences. Emotion regulation strategies are classified as “adaptive” or “maladaptive” based on their relationship with mental health difficulties (see Aldao et al., 2010 for review; Gross, 2015a). Maladaptive strategies often include rumination (focusing attention on distress), suppression (attempts to push away experience), and avoidance. Adaptive strategies include acceptance of experience, problem solving, and reappraisal of the meaning of experience.

The process model suggests that problems with emotion regulation occur at various stages, including the identification of the emotion and the selection, implementation, or modification of a strategy (Gross, 1998, 2015a, 2015b; Gross & Jazaieri, 2014). The extended version of this model includes the value placed on the affective experiences and how these impact the perceptions, goals, and actions of the person (Gross, 2015b).

Criticism of the process model often includes the limitations of judging specific strategies broadly as “adaptive” and “maladaptive” and the application of this to clinical interventions (Gratz et al., 2015). This is because the adaptiveness of strategies is often context-dependent and complex. Additionally, the inclusion of broad strategies (e.g., problem solving) risks subsuming multiple behaviors into the concept of emotion regulation, which may have different or multiple functions that may or may not be associated with emotion regulation (Gross, 2015a).

#### The Multidimensional Model of Emotion Regulation

In contrast, the multidimensional model (Gratz & Roemer, 2004) conceptualizes emotion regulation as global strategies employed when experiencing emotions. This model suggests that emotion regulation is made up of six domains: (1) the awareness and understanding of emotions, (2) acceptance of emotion-based experiences, (3) the ability to engage in goal-directed behaviors when distressed, (4) impulse control when experiencing emotions, (5) access to effective regulation strategies, and (6) willingness to experience difficult emotions as part of pursuing a meaningful life.

### The Development of Emotion Dysregulation in Gender and Sexual Minority Populations

Emotion regulation difficulties in GSM populations are thought to develop from the cumulative impact of minority and general stressors, alongside other vulnerability factors (Camp, 2023; Cardona et al., 2022; Drescher et al., 2020; Hatzenbuehler, 2009; Miu et al., 2022; Pachankis & Clark, 2025; Singh et al., 2023). GSM people are disproportionately exposed to childhood adversity and general stressors (e.g., childhood sexual and physical abuse) in addition to minority-specific stress, compounding the emotional burden (Jonas et al., 2022; Pachankis & Clark, 2025; Schneeberger et al., 2014; Solberg et al., 2023). While minority-specific stress alone explains some variance in the observed mental health disparity, other factors—such as adverse childhood experiences—may further explain these outcomes in relation to and separate from stigma-related stressors (Link & Phelan, 2001; Oshea et al., 2024; Pachankis & Clark, 2025; Pellicane & Ciesla, 2022; Solberg et al., 2023; Wilson et al., 2023).

Increased stress exposure interferes with accessing effortful adaptive emotion regulation strategies (e.g., problem solving and acceptance) and instead prompts the use of easier but maladaptive strategies to achieve shorter-term relief and to prevent further harm (e.g., avoidance and suppression; Cardona et al., 2022; Hatzenbuehler, 2009; Linehan, 1993; Link & Phelan, 2001; McCabe et al., 2021; Major & O’Brien, 2005; Singh et al., 2023). A lack of emotional validation and support from others, often observed in GSM populations, may further hinder adaptive skill development and reinforce internalized beliefs that emotions are unmanageable or unacceptable (Camp, 2023; Cardona et al., 2022; Drescher et al., 2023; Hatzenbuehler, 2009; Linehan, 1993).

Additionally, stigma-related proximal factors, like stigma sensitivity, internalized cis-heterosexism, vigilance for threat, and concealment of identity, may also undermine adaptive emotion regulation development (Doyle & Molix, 2015; Hatzenbuehler, 2009; Link & Phelan, 2001; Major & O’Brien, 2005; Pachankis, 2007; Puckett et al., 2015; Singh et al., 2023). For example, identity concealment and threat vigilance may heighten emotional suppression and thus reduce clarity, while internalized stigma may reinforce self-critical thinking and emotion avoidance. Thus, emotion regulation difficulties may serve both as a mechanism through which minority stress exerts its impact, and a more general risk factor that disproportionately affects GSM individuals due, in part, to their elevated exposure to multiple forms of adversity (Fraser et al., 2018; Girouard et al., 2021; Link & Phelan, 2001; Pachankis & Clark, 2025).

### Specific Domains of Emotion Regulation and Within-Group Heterogeneity

Research on specific domains of emotion regulation (e.g., emotion awareness and suppression) has shown varied associations with minority stress and mental health outcomes in GSM populations (Kaniuka et al., 2021; Mata-Greve et al., 2022; McLafferty et al., 2020). Given that some components of emotion regulation appear to mediate these relationships more strongly than others, particular strategies may play a more central role in the development of mental health difficulties than others (Gross, 2015a, 2015b; Gross & Jazaieri, 2014). Thus, minority stress, and other causal mechanisms, may have differing effects on specific emotion regulation strategies. It therefore may be that some specific emotion regulation processes are more important to consider for understanding mental health inequalities in GSM populations.

Additionally, research often collates sexual and gender minority groups together when exploring the risk factors for mental health outcomes. However, there is evidence for heterogeneity between GSM subgroups in these factors and thus a more nuanced understanding of within-GSM-group differences of emotion regulation is needed (Feinstein & Dyar, 2017; Hoy-Ellis, 2023; Rimes et al., 2020; Ross et al., 2018a, 2018b; Wittgens et al., 2022). This often includes differences between bisexual/pansexual (i.e., plurisexual) and gay/lesbian groups (i.e., monosexual minority groups), and transgender+ and cisgender groups, due to unique and overlapping stressors (Feinstein & Dyar, 2017; Hendricks & Testa, 2012; Reisner et al., 2023; Ross et al., 2018a, 2018b; Wittgens et al., 2022).

### The Current Study

Emotion regulation has been identified as a key transdiagnostic mechanism in the development and maintenance of mental health difficulties for GSM populations. Understanding which domains of emotion regulation difficulties are more prevalent in GSM individuals, and how these may contribute to mental health disparities when compared to cisgender-heterosexual people, is important to inform targeted interventions and efforts to reduce inequalities (Gillikin et al., 2021; Mata-Greve et al., 2022).

While the causes of emotion dysregulation—and their role in mediating the relationship between minority stress and mental health outcomes—are also important, these pathways have been well documented in the literature (Drescher et al., 2023; Hatzenbuehler, 2009; Mann et al., 2022; Mata-Greve et al., 2022) and synthesized in previous reviews (e.g., Singh et al., 2023). In contrast, this review focuses on emotion regulation difficulties as a potential independent or overarching risk factor contributing to mental health difficulties in GSM population compared to cisgender-heterosexual populations, rather than solely as a mechanism linking minority stress to mental ill health.

Currently, no systematic review or meta-analysis has explored whether GSM groups have higher global or specific emotion regulation difficulties compared to cisgender and heterosexual groups, nor whether such difficulties mediate the relationship between GSM identity and mental health outcomes to understand the role emotion regulation plays in health disparities between groups. This is an important gap, particularly as general psychological risk factors—such as emotion dysregulation—may disproportionately affect GSM populations despite not being exclusive to their minority status (Fraser et al., 2017; Girouard et al., 2021). Therefore, this review aimed to synthesize the current research regarding the following questions:

1. Do GSM people have higher levels of global and specific aspects of emotion dysregulation than cisgender and heterosexual groups?
2. Are there any within-group differences in levels of global and specific aspects of emotion dysregulation?
3. Do differences in emotion regulation processes mediate the relationship between sexual orientation/gender identity and psychological distress/mental health outcomes?

## Method

### Search Strategy

A systematic literature search (PROSPERO ref: CRD42023406448) was conducted following PRISMA guidelines (Moher et al., 2015). Searches were conducted through OvidSP using Embase (1947 to the date of search), Ovid MEDLINE® (1946 to the date of search), Global Health (1973 to the date of search), and APA PsychArticles and PsychInfo (1806 to the date of search). The search terms and Boolean operators are detailed in the Supplementary Materials. The search was applied to abstracts, keywords, and article titles. Reference lists of the full-text review articles and any meta-analyses/systematic reviews were hand-searched. The initial search was completed in June 2023 and updated in March 2024.

### Selection Criteria

Studies were included if they:

1. Included one or more clearly defined and/or stratified GSM group;
2. Reported quantitative data comparing emotion (dys)regulation between GSM groups or GSM and non-GSM groups, and/or reported mediation analyses with emotion (dys)regulation as a mediator, GSM identity as a predictor, and mental health outcomes as the dependent variables;
3. Were published in peer-reviewed journals;
4. Were available in English.

**Fig. 1:**
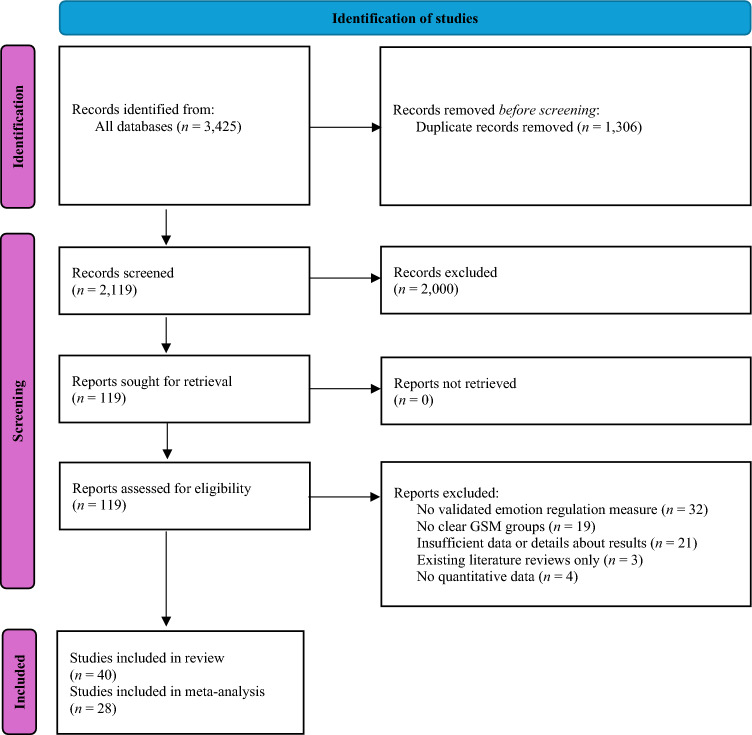
PRISMA (2020) flow diagram

The number of studies that met these criteria is described in Fig. 1.

Eligible studies assessed either global and/or specific validated measures of emotion regulation in line with the multidimensional model of emotion dysregulation (Gratz & Roemer, 2004) and the process model of emotion regulation (Gross, 1998, 2015a). We included specific strategies outlined in the process model (e.g., suppression and rumination; Gross, 1998, 2015a) only when they were explicitly conceptualized as emotion regulation strategies and with an intended regulation function, based on the measure used. Consistent with Gross (2015a), specific strategies (e.g., problem solving) were excluded if they served broader functions that were not measured as having an explicit association with or intention to regulate emotions. We used this approach to maximize construct validity and to avoid inclusion of overlapping constructs that may not be specific to regulating emotions. Additionally, we included alexithymia measures as a proxy for difficulties with emotion awareness, due to its definitional and measurement-item overlap with this specific domain of emotion regulation.

Mental health outcomes suitable for inclusion were validated measures of diagnosable disorders, symptom clusters, or overall psychological distress or well-being, and self-harm and suicidal behaviors. We excluded studies using only qualitative methodology or that did not disaggregate GSM groups. No restrictions were placed on participant age, sample size, or publication year.

#### Meta-Analysis Inclusion Criteria

Studies were eligible for inclusion in the meta-analysis if they reported the means, standard deviations, and sample sizes for relevant groups. Where such data were missing, corresponding authors were contacted. If data could not be retrieved, studies were retained for narrative synthesis if they met all other inclusion criteria. Where subgroup comparisons were possible but not reported in the studies, authors were invited to share disaggregated data.

### Selection Process

Duplicates were removed first by OVIDSP and then by EndNote. The first author screened the remaining titles, abstracts, and keywords against the inclusion criteria. Twenty percent of abstracts selected randomly were also screened by the second author, who was blind to the first author’s selections, to check for discrepancies. The full texts of potentially relevant articles were retrieved and screened against the eligibility criteria by both the first and second authors, who were blind to each other’s ratings.

### Data Extraction

Information extraction included the study citation; study design and location; sample size and characteristics; emotion regulation and mental health outcomes; and relevant analyses and results. Sociodemographic information was also extracted. GSM groups described as “other” were included under this designation. The mean, standard deviation, and sample size for each group were extracted where available.

### Quality Assessment

Study quality was assessed using the AXIS tool for observational studies (Downes et al., 2016). This consists of 20 items comprising five domains of study design and possible bias. Example items are “was the study design appropriate for the stated aim(s)” and “were the risk factor and outcome variables measured appropriate to the aims of the study.” Answers were rated as *yes* (1), *no* (0), or *unable to determine* (0) with a score range of 0–20. Higher scores indicate higher methodological quality and lower risks of bias. Authors JC and EB completed the AXIS tool for all included studies, masked to one another’s rating. Discrepant ratings were resolved through discussion. Consensus ratings for methodological quality are reported in Table 1.

**Table 1:** Consensus AXIS Tool methodological quality ratings

Study/References	Quality of reporting	Design quality	Risk of bias	Total
Barnett et al. (2022)	7.00	5.00	3.00	15.00
Bayer et al. (2017)	7.00	5.00	3.00	15.00
Bezahler et al. (2024)	7.00	5.00	2.00	14.00
Brotto et al. (2015)	7.00	5.00	3.00	15.00
Chang et al. (2020)	6.00	6.00	3.00	15.00
Chang et al. (2023)	7.00	5.00	2.00	14.00
Cao et al. (2023)	7.00	5.00	2.00	14.00
Drescher et al. (2023)	7.00	5.00	2.00	14.00
Dyar et al. (2024)	7.00	3.00	1.00	11.00
Eadeh et al. (2023)	6.00	4.00	2.00	12.00
English et al. (2018)	7.00	5.00	4.00	16.00
Fraser et al. (2018)	7.00	6.00	3.00	16.00
Gardella et al. (2021)	6.00	5.00	2.00	13.00
Gilkin et al. (2021)	7.00	5.00	3.00	15.00
Girouard et al. (2021)	7.00	5.00	3.00	15.00
Hatzenbeuhler et al. (2008)	7.00	5.00	3.00	15.00
Kaniuka et al. (2021)	7.00	5.00	2.00	14.00
Kapatais et al. (2023)	7.00	5.00	3.00	15.00
Kirwan et al. (2023)	7.00	5.00	3.00	15.00
Lattanner et al. (2022)	7.00	6.00	3.00	16.00
Lopez et al. (2022)	7.00	5.00	4.00	16.00
Mata-Greve et al. (2022)	7.00	5.00	2.00	14.00
Mazzoli et al. (2022)	3.00	2.00	1.00	6.00
McCabe et al. (2021)	7.00	5.00	4.00	16.00
McLafferty et al. (2020)	6.00	5.00	2.00	13.00
Ockerman et al. (2025)	7.00	5.00	2.00	14.00
Pachankis et al. (2015)	6.00	4.00	3.00	13.00
Petruzzelli (2022)	7.00	5.00	3.00	15.00
Ramos et al. (2022)	7.00	6.00	5.00	18.00
Reitzel et al. (2017)	7.00	5.00	2.00	14.00
Rogers et al. (2017)	6.00	5.00	3.00	14.00
Schroeder et al. (2024)	7.00	5.00	2.00	14.00
Servaty-Seib et al. (2023)	7.00	5.00	4.00	16.00
Silveri (2021a, 2021b)	7.00	5.00	3.00	15.00
Sommantico and Parrello (2022)	7.00	5.00	2.00	14.00
Teixeira (2022)	5.00	6.00	2.00	13.00
Ugueto and Lucassen (2022)	7.00	6.00	4.00	17.00
Vogel et al. (2024)	7.00	5.00	5.00	17.00
Warmuz-Stangier (2015)	6.00	3.00	2.00	11.00
Descriptive Statistics				
*M*	6.67	4.92	2.74	14.33
SD	0.77	0.81	0.94	2.02
Min	3.00	2.00	1.00	6.00
Max	7.00	6.00	5.00	18.00

*Note.* Quality of Reporting Score: items 1, 4, 10, 11, 12, 16, 18; Study Design Quality: items 2, 3, 5, 8, 17, 19, 20; Attempts to reduce bias: items 6, 7, 9, 13, 14, 15; Total = total score: all items

### Data Synthesis and Analysis

Studies lacking sufficient data for the meta-analysis were analyzed using a narrative synthesis only. The narrative synthesis also included findings from studies where group comparisons or outcomes were not included in other studies for comparison.

Meta-analyses were conducted in STATA (V18; *meta esize*) where adequate data were available or provided upon request. We examined four comparison pairs based on the most commonly used groupings: (1) sexual minorities compared to heterosexual people; (2) sexual and gender minorities compared to cisgender-heterosexual people where studies did not disaggregate further; (3) bi+/plurisexual (people with attractions to multiple sexes and genders, or identifying as bisexual, pansexual or similar) compared to gay+/monosexual minority people (people with predominately same-sex or –gender attractions, or who identify as gay, lesbian, or similar); and (4) transgender+groups compared to cisgender groups.

Means, standard deviations, and sample sizes were extracted. Hedges *g* was generated for each study and meta-analyzed (*meta summarize*) using the random effects model, restricted maximum likelihood method (*reml*), to account for heterogeneity. Measures of between-study heterogeneity (Cochran’s *Q*: *I*^2^%) were produced, and results were pooled into forest plots (*meta forest plot*). Heterogeneity over 50% was considered high (Higgins et al., 2024). We conducted a post-hoc sensitivity analysis to test for comparisons involving the most commonly used measures (i.e., DERS or ERQ) to assess their impact on heterogeneity and pooled effect sizes (Higgins et al., 2024).

## Results

The search identification, screening, and inclusion results are presented in Fig. 1.

### Study Characteristics

See Table 2 for study characteristics. The total number of participants across the included studies was *N* = 24,649. The average age across the studies was 26.03 years (8.64) with a range of averages from 12.56 to 53.37 years. Eleven studies included children and/or adolescent samples. Sixty-two percent of the participants were heterosexual and 38% were sexual minorities. Where this was further disaggregated (not all studies provided disaggregation of sexual minority identities and thus percentages differ slightly from above), 1% were asexual, 15% were in the plurisexual/bi+ group, 13% were in the gay/lesbian/monosexual minority group, 67% were heterosexual, 3% were included as “other,” and less than 1% were queer or questioning/unsure, respectively.

**Table 2:** Sample characteristics and methodology of the included studies

Study	Sample characteristics					Methodology			
	Sexual orientation	Gender	Sex assigned at birth	Race/Ethnicity	Age in years *M *(SD)	Country of origin	Sample method and source	Emotion regulation variable and measure	*N*
Barnett et al. (2022)	31% Bisexual 6% Gay/Lesbian/Queer 57% Heterosexual 7% Undecided	100% Cisgender Female	100% Female	5% American Indian/Alaskan Native 1% Asian 20% Black or African American 48% Hispanic/Latinx 19% Multiracial 1% Native Hawaiian/Pacific Islander 23% Other 31% White	15.58 (1.10)	USA	Convenience High schools; juvenile intake department for a family court; community agencies; study staff attending appointments at courthouse	Global Emotion Regulation: Affect Dysregulation Scale (Brown et al., 2012)	226
Bayer et al. (2017)	48% Bisexual 52% Lesbian	100% cisgender Female	100% Female	4% African American 2% Asian American/Pacific Islanders 72% Caucasian 7% Latina 10% Multi/Biracial	Bisexual: 29.30 (9.80) Lesbian: 35.00 (13.60)	USA	Convenience SM + organizations; university pool	Global Emotion Regulation: Distress Tolerance Scale (Simons & Gaher, 2005)	138
Bezahler et al. (2024)	78% heterosexual 22% Sexual Minorities: (6% Gay/Lesbian, 8% Bi + , 3% Asexual, 2% Queer, 3% Other Orientation)	45.30% Cisgender Men 51% Cisgender Women 2% Gender Nonconforming < 1% Nonbinary < 1% Not Listed < 1% Transgender +	NS	2% African American/Black 4% Asian 3% Don’t know 4% Latin/x < 1% Native American/Alaskan 5% Not listed 80% White	29.20 (10.70)	USA	Convenience Partial-hospital/residential treatment program	Global Emotion Regulation and Emotion Acceptance, Emotion Clarity, Goal-Directed Behavior, Impulse Control, Strategies Subscales: Difficulties in Emotion Regulation Scale, Short Form (Kaufman et al., 2016)	470
Brotto et al. (2015)	Asexual Group (via the AIS > 40): 78% Asexual 4% Bisexual 16% Heterosexual 2% Homosexual Not Asexual Group (AIS < 40): 2% Asexual 18% Bisexual 71% Heterosexual 9% Homosexual	NS	Asexual: 79.7% Female, 20.3% Male Not Asexual: 68.1% Female*, 31.9% Male	Asexual: < 1% African American 4% East/South Asian 91% Euro-Caucasian 2% Hispanic 3% Other Not Asexual: 4% African American 10% East/South Asian 80% Euro-Caucasian 2% Hispanic 5% Other	Asexual: 30.90 (11.10) Not Asexual: 33.90 (12.30)	Canada	Convenience Postings on local websites; web-community discussion boards; online and in-clinic postings at offices of sex therapists and sexologists; university pool	Emotion Awareness: The Toronto Alexithymia Scale (Bagby et al., 1994)	424
Chang et al. (2020)	4% Asexual 38% Bisexual/Pansexual 1% Did Not State 22% Gay/Lesbian 33% Heterosexual 2% Other	64% Cisgender Female 29% Cisgender Male 6% Gender Queer/Nonconforming < 1% Other < 1% Transfemale/Women < 1% Transmale/men	NS	11% African American 16% Asian 12% Hispanic/Latinx 2% Native American 8% Other/Multiracial 51% White	25.41 (9.36)	USA	Convenience In-person sample: flyers and advertisements, university pool online sample: Amazon Mechanical Turk (MTurk)	Global Emotion Regulation: Difficulties in Emotion Regulation Scale (Gratz & Roemer, 2004)	388
Chang et al. (2024)	18% Sexual Minorities, of which: 42% Bisexual 5% Gay/Lesbian 24% Not Sure 30% Something Else	46% Cisgender Boys 53% Cisgender Girls 1% Other	54% Female 46% Male	4% Asian 12% Black 9% Hispanic/Latinx 7% Multiracial < 1% Native American 2% Other or No Report 74% White	15.90 (1.09)	USA	Convenience Commercial mailing lists, online and community advertisements, and ResearchMatch	Global Emotion Regulation: Difficulties in Emotion Regulation Scale (Gratz & Roemer, 2004)	241
Cao et al. (2023)	NS	70% Transgender + Females 30% Transgender + Males	70% Male 30% Female	NS	24.57 (6.42)	China	Snowball Peer-driven sampling via an initial set of participants and then people they knew	Global Emotion Regulation: Difficulties in Emotion Regulation Scale (Gratz & Roemer, 2004)	971
Drescher et al. (2023)	21% Bisexual < 5% Gay, Lesbian, Queer, Questioning, or Other 12% Heterosexual 24% Pansexual	100% Transgender + or Gender Nonconforming	NS	< 2% Asian, American Indian/Alaskan Native, or Hispanic 11% Black/African American 20% Did not answer 9% Multiracial 57% White	27.61 (9.38)	Georgia	Convenience From a specialist GSM + clinic	Global Emotion Regulation: Difficulties in Emotion Regulation Scale, Short Form (Kaufman et al., 2016)	115
Dyar (2024)	26% Bisexual 26% Lesbian 26% Pansexual 22% Queer	73% Cisgender Women 27% Gender Minority	100% Female	12% Asian 24% Black 30% Latinx 8% Other 55% White	22.27 (2.01)	USA	Convenience Paid advertisement via social media	Reappraisal and Emotion Suppression: Daily Diary of Global Emotion Regulation Strategies	429
Eadeh et al. (2023)	3% Another Orientation 10% Asexual 34% Bisexual 3% Demisexual 10% Gay 13% Heterosexual 12% Lesbian 13% Pansexual 3% Queer < 1% Questioning	3% Cisgender Men 8% Cisgender Women 20% Nonbinary or Gender Fluid 32% Transgender + Man 37% Transgender + Woman	43% Male 57% Female < 1% Intersex	1% American Indian/Alaska Native 5% Another race 1% Asian 4% Black/African American 5% Hispanic 89% White	27.84 (11.30)	USA	Convenience GSM + Counseling Clinic	Global Emotion Regulation and Emotion Awareness, Emotion Acceptance, Emotion Clarity, Goal-Directed Behavior, Impulse Control, Strategies Subscales: Difficulties in Emotion Regulation Scale, Short Form (Kaufman et al., 2016)	155
English et al. (2018)	15% Bisexual 85% Gay, Queer, or Homosexual	100% Cisgender Men	NS	43% Black 30% Latino 25% Multiracial	35.45 (10.45)	USA	Convenience, Snowball Respondent-driven, internet advertisements, emails through gay sex party listservs, active recruitment in gay bars/clubs and sexual minority community events	Global Emotion Regulation: Difficulties in Emotion Regulation Scale (Gratz & Roemer, 2004)	170
Fraser et al. (2018)	< 1% Asexual 3% Bisexual 89% Heterosexual < 1% Homosexual 6% Mostly Heterosexual < 1% Mostly Homosexual	57% Cisgender Female 43% Cisgender Male < 1% Transgender +	NS	78% New Zealand European/Pakeha 6% Maori 3% Samoan 10% Other	15.16 (2.61)	New Zealand	Convenience Existing longitudinal study dataset (Youth Wellbeing Study) which recruited from sixteen different schools	Global Emotion Regulation: Emotion Regulation Index for Children and Adolescents (MacDermott et al., 2010)	1,799
Gardella et al. (2021)	35% Feminine Sexual Minority Women 34% Heterosexual 31% Masculine Sexual Minority Women	31% Masculine-Presenting Cisgender Females 69% Feminine-Presenting Cisgender Females	NS	10% Asian/Pacific Islanders 13% Black/Black American/Caribbean 12% Hispanic/Latinx 10% Multiracial 1% Other 54% White/European	24.58 (3.01)	USA	Convenience, Snowball Craigslist, professional email listservs, flyers in targeted locations	Emotion Suppression and Reappraisal: Emotion Regulation Questionnaire (Gross & John, 2003)	147
Gillikin et al. (2021)	50% Heterosexual 50% Lesbian, Gay, Bisexual, of which: 67% Bisexual, 28% Gay/Lesbian, 6% Other	NS	Sexual Minorities: 66% Female, 34% Male Heterosexual: 60% Female, 40% Male	Sexual Minorities: 1% American Indian/Alaskan Native/First Nations, 3% Asian, 5% Black/African, 9% Hispanic/Latinx, 1% Other, 90% White/Caucasian Heterosexual: 2% American Indian/Alaskan Native/First Nations, 13% Asian, 16% Black/African American, 13% Hispanic/Latinx, 4% Other, 64% White/Caucasian	Sexual Minority: 33.00 (9.69) Heterosexual: 38.00 (11.97)	USA	Convenience Amazon Mechanical Turk (MTurk)	Emotion Awareness and Emotion Acceptance and Goal-Directed Behavior, Impulse Control, Strategies Subscales: Difficulties in Emotion Regulation Scale (Gratz & Roemer, 2004)	388
Girouard et al. (2021)	5% Bisexual 1% Gay/Lesbian 1% Did Not Answer 1% Heteroflexible 85% Heterosexual < 1% Homoflexible 3% None of These 2% Pansexual < 1% Queer 3% Questioning	< 1% Bi-Spiritual 49% Boy 50% Girl < 1% Nonbinary/Gender Fluid < 1% Other Trans Status: < 1% Does Not Know What Trans Means 99% Not Trans < 1% Questioning	49% Male 51% Female	12% Canadian Culture 17% Other Cultural Identities 71% Quebecois Culture	14.60 (0.60)	Canada	Convenience Schools	Global Emotion Regulation: Difficulties in Emotion Regulation Scale, Short Form (Kaufman et al., 2016)	1,036
Hatzenbuehler et al. (2008)	98% Heterosexual 2% Sexual Minority	49% Females 51% Males	NS	2% Asian/Pacific Islander 9% Biracial or Multiracial 57% Hispanic/Latino < 1% Middle Eastern < 1% Native American 13% Non-Hispanic Black 4% Other	NS: 32% Sixth Grade of School, 34% Seventh Grade, 34% Eighth Grade	USA	Convenience Schools	Rumination: Children's Response Styles Questionnaire (Abela et al., 2002) Emotion Awareness: Emotion Expression Scale for Children (Penza-Clyve & Zeman, 2002)	1,065
Kaniuka et al. (2021)	16% Bisexual 8% Lesbian/Gay 65% Heterosexual 11% Other	60% Cisgender Female 38% Cisgender Male 2% Other	NS	34% Other 66% White	31.17 (13.34)	USA	Convenience Amazon Mechanical Turk (MTurk), college campus, advocacy organization for SM + members listserv	Emotion Suppression and Reappraisal: Emotion Regulation Questionnaire (Gross & John, 2003)	2,125
Kapatais et al. (2023)	64% Heterosexual 36% GSM + (3% Asexual, 20% Bisexual, 3% Gay, 3% Lesbian, 2% Pansexual, 6% Queer or Questioning)	76% Female 22% Male 2% Prefer Not to Say 98% Cisgender 2% Transgender +	NS	1% African American 3% Asian 5% Mixed Race 3% Other 88% White	24.15 (8.49)	UK	Convenience University, social media	Global Emotion Regulation: Difficulties in Emotion Regulation Scale (Gratz & Roemer, 2004)	484
Kirwan et al. (2023)	68% Heterosexual 31% Sexual Minority 34% Cisgender-Heterosexual Men 34% Cisgender-Heterosexual Women 31% Sexual-Gender Minorities	57% Cisgender Female 41% Cisgender Male 2% Other	NS	10% Asian/Pacific Islander 17% Hispanic/Latinx* 9% Multiracial 43% White	19.13 (1.09)	USA	Random Population University	Global Emotion Regulation: Difficulties in Emotion Regulation Scale (Gratz & Roemer, 2004)	754
Lattanner et al. (2022)	26% Bisexual 74% Gay	97% Cisgender Male < 1% Nonbinary 1% Other 1% Transgender + Male	NS	< 1% American Indian or Alaska Native 2% Asian 7% Black or African American < 1% Don't Know 12% Hispanic/Latino < 1% Native Hawaiian or other Pacific Islander 2% Other < 1% Refused 77% White	53.37 (14.79)	USA	Stratified Population Ipsos KnowledgePanel	Emotional Clarity: Difficulties in Emotion Regulation Scale, Short Form, Clarity Subscale (Kaufman et al., 2016) Rumination: The Response Styles Questionnaire (Treynor et al., 2003	502
Lopez et al. (2022)	80% Heterosexual 20% Lesbian, Gay, Bisexual	NS	NS	3% Asian 4% Biracial 5% Black 10% Hispanic < 1% Pacific Islander 6% Unknown 75% White	12.56 (0.60)	USA	Convenience Flyers and mailings	Global Emotion Regulation: Difficulties in Emotion Regulation Scale (Gratz & Roemer, 2004)	177
Mata-Greve et al. (2022)	36% Bisexual 11% Gay or Lesbian 6% Other Sexuality 47% Straight	1% Gender Diverse 34% Men 7% Nonbinary 1% Transgender + 57% Women	NS	13% African American/Black 2% American Indian and Alaska Native 4% Another Race 21% Asian American 49% European American 11% Multiracial	27.90 (7.60)	USA	Convenience Prolific research platform	Global Emotion Regulation and Emotion Awareness, Emotion Acceptance, Emotion Clarity, Goal-Directed Behavior, Impulse Control, Strategies Subscales: Difficulties in Emotion Regulation Scale, Short Form (Kaufman et al., 2016)	417
Mazzoli et al. (2022)	NS	29% Cisgender Men 22% Cisgender Women 26% Transgender + Men 23% Transgender + Women	NS	NS	NS	Italy	Convenience Gender clinics	Emotion Awareness: The Toronto Alexithymia Scale (Bagby et al., 1994)	789
McCabe et al. (2021)	90% Heterosexual/Straight 10% Sexual Minorities, of which: 74% Bisexual, 26% Lesbian	100% Cisgender Female	NS	53% Black 6% Other/Multiracial 41% White	Range 17–22	USA	Stratified/random, with oversampling of households in low-income neighborhoods in a particular state Household sampling	Emotion Awareness and Strategies Subscale: Difficulties in Emotion Regulation Scale (Gratz & Roemer, 2004) Rumination: Perfectionism Inventory: Subscale Rumination (Hill et al., 2004)	2,447
McLafferty et al. (2020)	90% Heterosexual 9% Non-Heterosexual	63% Female 37% Male	NS	NS	20.69 (5.31)	UK & Republic of Ireland	Convenience University	Emotion Suppression and Reappraisal: Emotion Regulation Questionnaire (Gross & John, 2003)	739
Ockerman et al. (2025)	NS	10% Assigned Female to Nonbinary 6% Assigned Male to Nonbinary 45% Cisgender 21% Transgender + Men 19% Transgender + Women	58% Female 42% Male	< 1% American Indian < 1% Asian 8% Black < 1% Hawaiian/Pacific 17% Hispanic < 1% Multiracial 90% White	35.80 (18.00)	USA	Convenience Amazon MTurk	Global Emotion Regulation: five-item Difficulties in Emotion Regulation Scale (Bardeen et al., 2012)	337
Pachankis et al. (2015)	12% Bisexual 88% Gay, Queer, Homosexual	100% Male (Inclusive of Cisgender and Transgender +)	NS	2% Asian/Pacific Islander 20% Black 14% Latino 14% Other/Multiracial 51% White	36.90 (11.40)	USA	Convenience Respondent-driven, internet advertisements, emails through gay sex party listservs, active recruitment in gay bars/clubs and sexual minority community events	Global Emotion Regulation: Difficulties in Emotion Regulation Scale (Gratz & Roemer, 2004)	374
Petruzzelli et al. (2022)	NS	50% Cisgender 50% Gender Diverse	77% Female 23% Male	NS	15.60 (1.60)	Italy	Convenience Child Neuropsychiatry Unit, high schools	Global Emotion Regulation and Emotion Awareness, Emotion Acceptance, Emotion Clarity, Goal-Directed Behavior, Impulse Control, Strategies Subscales: Difficulties in Emotion Regulation Scale (Gratz & Roemer, 2004)	60
(Ramos et al., 2022)	82% Heterosexual	48% Boy 51% Girl < 1% Not Stated	52% Female 48% Male	NS	14.53 (0.61)	Canada	Stratified Population Schools	Global Emotion Regulation: Difficulties in Emotion Regulation Scale, Short Form (Kaufman et al., 2016)	2,904
Reitzel et al. (2017)	47% Bisexual 53% Monosexual (Minorities)	31% Cisgender Female 50% Cisgender Male 19% Transgender +	NS	64% Other 36% White	36.40 (14.80)	USA	Convenience Strategic flyer posting and paid mediums	Global Emotion Regulation: Distress Tolerance Scale (Simons & Gaher, 2005)	119
Rogers et al. (2017)	62% lesbian/gay 38% bisexual	53% Cisgender Female 47% Cisgender Male	NS	2% American Indian/Alaskan Native 5% Asian 3% Black/African American 2% Mixed Race < 1% Native Hawaiian/Pacific Islander 3% Other 84% White/Caucasian	28.68 (12.08)	USA	Convenience Facebook advertisements, Craigslist, emails to SM organizations and listservs	Global Emotion Regulation: Emotion Regulation Questionnaire (Gross & John, 2003)	305
Schroeder et al. (2024)	< 1% Asexual 8% Bisexual 66% Exclusively Heterosexual 3% Exclusively Homosexual 13% Mostly Heterosexual 2% Mostly Homosexual < 1% Other 3% Pansexual	97% Cisgender 3% Transgender +	64% Female	61% White	19.81 (3.35)	USA	Convenience University	Global Emotion Regulation and Emotion Awareness, Emotion Acceptance, Emotion Clarity, Goal-Directed Behavior, Impulse Control, Strategies Subscales: Difficulties in Emotion Regulation Scale Short Form (Kaufman et al., 2016)	701
Servaty-Seib et al. (2023)	87% Heterosexual 13% Underrepresented	64% Cisgender Female 35% Cisgender Male < 1% Transgender + Male < 1% Transgender + Female	NS	9% Asian 8% Biracial 3% Black 4% Hispanic/Latinx 2% Middle Eastern < 1% Native American < 1% Prefer not to answer 1% Self-specify 72% White	18.57 (0.50)	USA	Convenience University	Emotion Awareness and Global Emotion Regulation: Brief Emotional Intelligence Scale (Davies et al., 2010)	665
Silveri et al. (2021a, 2021b)	NS	82% Cisgender 18% Transgender + / Gender Nonconforming	55% Female 45% Male	2% African American/Black < 1% Alaskan Native/American Indian 2% Asian/Pacific Islander 5% Latinx/Hispanic 2% Multiracial 4% Other 92% White	16.20 (1.50)	USA	Convenience Patients admitted to short-term acute residential treatment for psychiatric disorders	Global Emotion Regulation: Difficulties in Emotion Regulation Scale (Gratz & Roemer, 2004)	200
Sommantico and Parrello (2022)	35% Bisexual 65% Gay	100% Cisgender Men	NS	NS	36.20 (11.60)	NS	Convenience, Snowball Adverts via social media and asking participants to recruit people they know	Global Emotion Regulation and Emotion Awareness, Emotion Acceptance, Emotion Clarity, Goal-Directed Behavior, Impulse Control, Strategies Subscales: Difficulties in Emotion Regulation Scale (Gratz & Roemer, 2004)	165
Teixeira et al. (2022)	43% Heterosexual 57% Non-Heterosexual	100% Men	NS	64% Non-White 36% White	NS	Brazil	Respondent-Driven Digital social networks	Emotion Suppression and Reappraisal: Emotion Regulation Questionnaire (Gross & John, 2003)	1,015
Ugueto and Lucassen (2022)	Females Sex: 32% Bisexual, 8% Gay or Lesbian, 42% Heterosexual/Straight, 8% Mostly Heterosexual/Straight, 2% Not Sure Yet, 6% Other, 2% Prefer Not to Answer Males Sex: 6% Bisexual, < 1% Do Not Understand, 4% Gay or Lesbian, 78% Heterosexual/Straight, 6% Mostly Heterosexual/Straight, < 1% Not Sure Yet, 2% Other, < 1% Prefer Not to Answer	Female Sex: < 1% Boy/Men, 92% Girl/Women, 3% Gender Queer, < 1% Other, < 1% Prefer Not to Say, 3% Trans Boy/Men Male Sex: 95% Boy/Men, 2% Don't Know, 2% Gender Queer, 2% Trans Girl/Woman	64% Female 36% Male	3% Asian/Asian American 19% Black/African American 40% Hispanic/Latinx 7% Other 14% Two or more races/ethnicities 17% White/Caucasian	15.31 (1.42)	USA	Convenience Acute child and adolescent unit in an inpatient, psychiatric hospital	Global Emotion Regulation: Difficulties in Emotion Regulation Scale, Short Form (Kaufman et al., 2016)	348
Vogel et al. (2024)	Women: 70% Bisexual 18% Lesbian 12% Other Orientation Men: 34% Bisexual 54% Gay 12% Other Orientation	58% Women 32% Men	NS	Women: 12% Another Race 8% Asian 4% Black 10% Hispanic Men: 10% Another Race 7% Asian 4% Black 13% Hispanic	Women: 24.11 (4.65) Men: 24.68 (4.48)	USA	Convenience Social media targeted advertisements	Emotion Suppression and Reappraisal: Emotion Regulation Questionnaire (Gross & John, 2003)	776
Warmuz-Stangierska et al. (2015)	NS	33% Cisgender Females 33% Cisgender Males 33% Transgender +	NS	NS	Cisgender Females: 25.75 (4.19) Cisgender Males: 26.07 (3.44) Transgender + : 24.93 (4.06)	Poland	Not stated Gender Identity Disorders Section at University of Medical Sciences	Emotion Suppression: Courtauld Emotional Control Sale (Watson & Green 1983)	84

Note. gender specified as transgender + or cisgender where this was present by the author or clear from the data. Where this was not clear, the author’s terminology is used, including gender without “trans” or “cis” prefixes. NS = not stated/described in the study. Race/ethnicity groups described as they were described in the study they are extracted from. *M* = mean and SD = standard deviation. *N* = sample size. USA = United States of America. UK = United Kingdom of Great Britain and Northern Ireland. SM = Lesbian, Gay, Bisexual and other minority stress orientations. GSM = Lesbian, Gay, Bisexual, Transgender + , Queer, and other minority gender identities and sexual orientations. The AIS Asexuality Identification Scale is a measure designed to distinguish asexual individuals from sexual individuals for research purposes (Yule et al., 2014)

Fifty-seven percent of the overall sample were female assigned sex and 43% were male. For gender identity, 87% were cisgender, and 13% transgender+. Where studies further disaggregated transgender+ groups (not all studies provided disaggregated data for transgender + groups and thus percentages differ slightly from above), 10% were binary transgender+, 89% were cisgender, 1% were nonbinary or gender queer, and less than 1% were described as “other.”

Regarding ethnicity/race, 3% were Asian, 12% were Black, 10% were Hispanic/Latinx, 4% were mixed ethnicity/multiracial, 2% were Native/Indigenous, 8% were described as “other,” and 61% were White. Overall, 39% of the samples were from minoritized ethnicity-/race-related backgrounds.

Most studies utilized convenience sampling methods and were conducted in the USA. Studies included a variety of emotion regulation constructs (see Table 3) measured with various measures of emotion dysregulation, with the most frequently being the Difficulties with Emotion Regulation Scale (DERS; Gratz & Roemer, 2004) and the Emotion Regulation Questionnaire (ERQ; Gross & John, 2003).

**Table 3:** Included emotion regulation constructs

Emotion Regulation Construct/Domain	Definition
Global Emotion Regulation	Global strategies employed when experiencing emotions often in the domains of emotion awareness and clarity, acceptance of emotions, perceived access to regulation strategies, and the ability to act in a goal-directed manner and to inhibit impulsive urges when experiencing emotions (Gratz & Roemer, 2004) A set of strategies to modify affective experiences in the domains of situation selection and modification, attentional deployment, cognitive change, and response modulation (Gross, 1998, 1999, 2015a, 2015b)
The Multidimensional Model (Gratz & Roemer, 2004)	
Emotion Awareness	The ability to attend to and acknowledge emotions
Emotional Clarity	The ability to understand and be clear about the emotions they are experiencing
Nonacceptance of Emotions*	Negative secondary emotions and nonacceptance of one’s emotions
Goal-Directed Behavior	Engaging in goal-directed behavior, such as concentrating or accomplishing tasks, when experiencing emotions
Impulse Control	Maintaining control over behaviors and urges when experiencing emotions
Access to Regulation Strategies	Perceived access to strategies to regulate emotions effectively when experiencing emotions
The Process Model (Gross, 1998, 1999, 2015a, 2015b)	
Reappraisal	Modifying the evaluation/appraisal of the emotion cue to decrease emotion arousal
Emotion Suppression	Suppression/inhibition of emotion expression and responses
Rumination	Excessive focus of attention on cues and states of emotions

Note. Not included: avoidance of emotion cues or experiencing and problem solving to change emotional arousal from the Process Model due to insufficient studies investigating these outcomes or measuring them as emotion regulation strategies

^*^Also included in the process model

### Meta-Analysis

Results from the meta-analyses are described in Figs. 2, 3, 4, 5, 6, 7, 8, 9, 10, and 11. The sensitivity analysis is also described in Table 4.

**Table 4:** Sensitivity analysis including consistent measures only

Comparison groups	Original meta-analysis						Meta-analysis with consistent measures only					
	Effect size		Heterogeneity				Effect size		Heterogeneity			
	*g*	95% CI	*Q*	df	*p*	*I*^*2*^	*g*	95% CI	*Q*	df	*p*	*I*^*2*^
*Global Emotion Regulation (DERS)*												
Sexual Minorities cf Heterosexual Groups	0.46	0.38, 0.54	19.45	11	.80	43.91%	0.41	0.35, 0.47	9.38	8	.31	0.00%
Plurisexual cf. Monosexual Minority Groups	0.23	0.11, 0.34	12.87	11	.30	0.00%	0.22	0.05, 0.38	11.98	7	.10	27.88%
Transgender + cf Cisgender Groups	0.40	0.15, 0.65	12.73	6	.05	55.02%	0.42	0.15, 0.68	6.43	3	.09	55.00%
*Emotion Awareness (DERS)*												
Sexual Minorities cf Heterosexual Groups	0.18	0.04, 0.32	24.34	7	< .01	76.79%	0.16	− 0.04, 0.36	16.56	4	< .01	83.77%
Plurisexual cf. Monosexual Minority Groups	0.20	0.06, 0.34	5.11	6	.53	0.00%	0.18	0.03, 0.34	4.24	4	.37	7.89%
Transgender + cf Cisgender Groups	0.25	− 0.08, 0.59	20.76	4	< .01	75.01%	0.12	− 0.27, 0.50	0.11	1	.74	0.00%
*Cognitive Reappraisal (ERQ)*												
Plurisexual cf. Monosexual Minority Groups	0.01	− 0.11, 0.21	0.68	2	.71	0.00%	0.04	− 0.12, 0.19	0.35	1	.56	0.00%
*Emotion Suppression (ERQ)*												
Plurisexual cf. Monosexual Minority Groups	− 0.07	− 0.21, 0.06	1.09	2	.58	0.00%	− 0.11	− 0.27, 0.04	0.08	1	.77	0.00%

*Note.* Random Effects REML Model. Emotion regulation measures: DERS = Difficulties in Emotion Regulation Scale (Gratz & Roemer, 2004); ERQ = Emotion Regulation Questionnaire (Gross & John, 2003)

#### Global Emotion Regulation

**Fig. 2:**
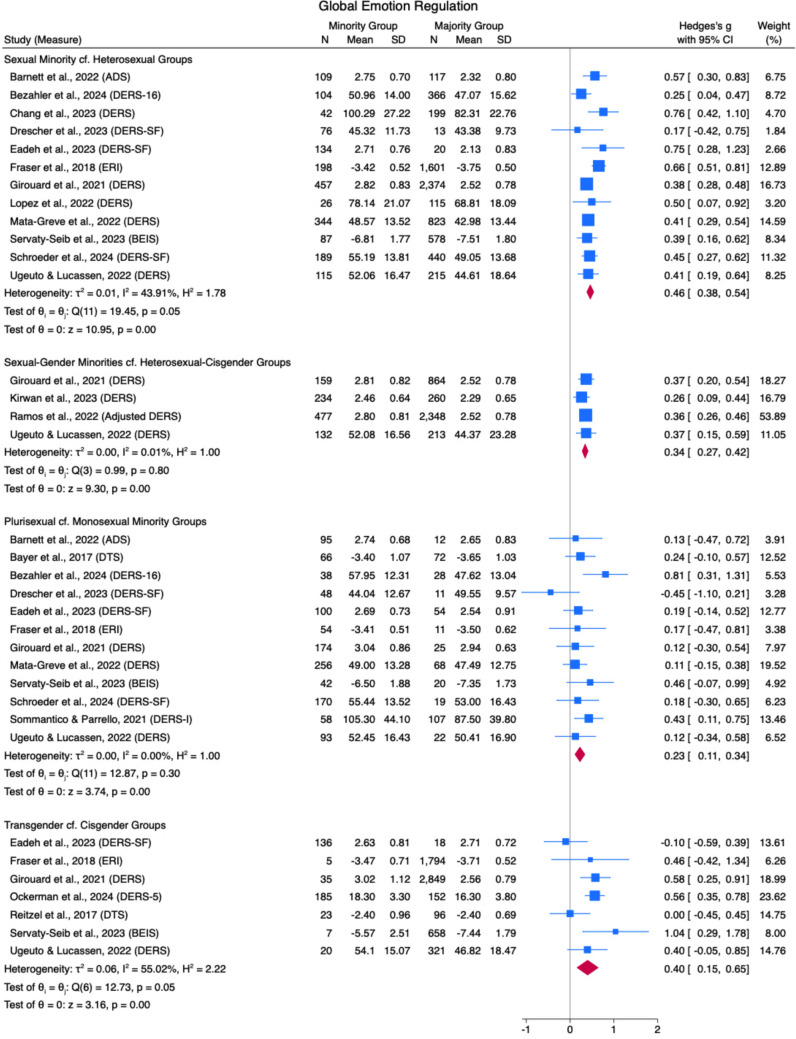
Global Emotion Regulation Forest Plot. *Note.* Random Effects REML Model. *N* = sample size. *M* = mean. SD = standard deviation. Emotion regulation measures: ADS = Affect Dysregulation Scale (Brown et al., 2012); BEIS = Brief Emotional Intelligence Scale (Davies et al., 2010); DERS = Difficulties in Emotion Regulation Scale (Gratz & Roemer, 2004); DERS-5 = five-item DERS (Bardeen et al., 2012); DERS-SF/16 = DERS Short Form (Kaufman et al., 2016); DTS = Distress Tolerance Scale (Simons & Gaher, 2005)

Twelve studies suggested that sexual minority populations scored higher on global emotion dysregulation than heterosexual groups, with a small pooled effect size (*g* = 0.46 [0.38, 0.54]; see Fig. 2) and moderate heterogeneity (*Q*[11] = 19.45, *p* = .05, *I*^*2*^ = 43.91%). In four studies, GSM people scored higher than cisgender-heterosexual people on global emotion dysregulation, with a small pooled effect size (*g* = 0.34 [0.27, 0.42]) and low heterogeneity between studies (*Q*[3] = 0.99, *p* = .80, *I*^*2*^ = 0.01%; see Fig. 2). Plurisexual groups scored higher than monosexual minority groups on measures of emotion dysregulation across 12 studies, with a small pooled effect size (*g* = 0.23 [0.11, 0.34]) and low heterogeneity (*Q*[11] = 12.87, *p* = .30, *I*^*2*^ = 0.00%). Seven studies found that transgender + groups scored higher on measures of emotion dysregulation compared to cisgender groups, with a small pooled effect size (*g* = 0.40 [0.15, 0.65]) and high heterogeneity (*Q*[6] = 12.73, *p* = .05, *I*^*2*^ = 55.02%).

#### Emotion Awareness

**Fig. 3:**
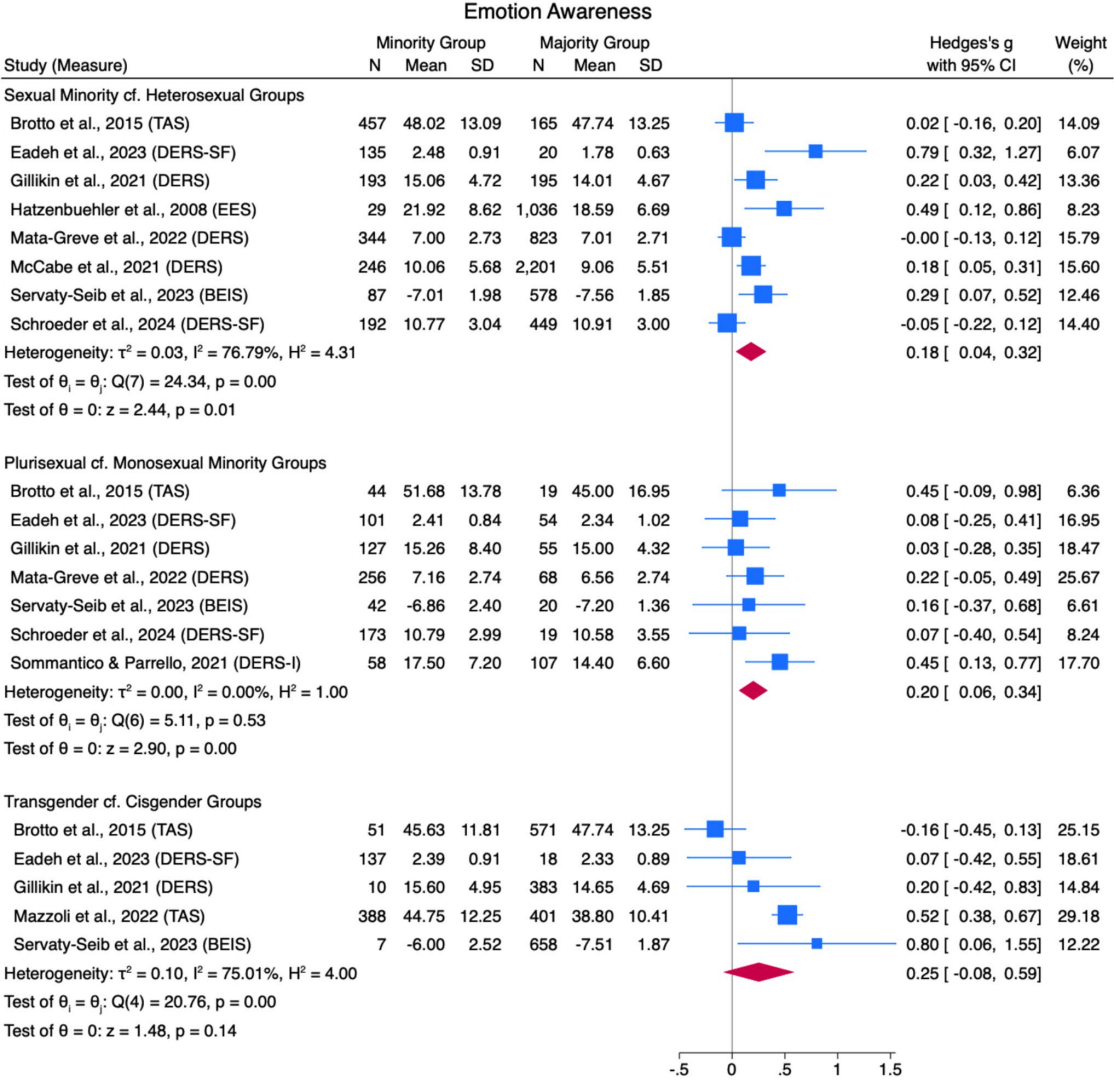
Emotion Awareness Forest Plot. *Note.* Random Effects REML Model. *N* = sample size. *M* = mean. *SD* = standard deviation. Emotion regulation measures: ADS = Affect Dysregulation Scale (Brown et al., 2012); BEIS = Brief Emotional Intelligence Scale (Davies et al., 2010); DERS = Difficulties in Emotion Regulation Scale (Gratz & Roemer, 2004); DERS-5 = five-item DERS (Bardeen et al., 2012); DERS-SF = DERS Short Form (Kaufman et al., 2016); EES = Emotion Expression Scale for Children (Penza-Clyve & Zeman, 2002); TAS = The Toronto Alexithymia Scale (Bagby et al., 1994)

Sexual minorities scored higher on measures of emotion awareness difficulties than heterosexual groups, with a below-small pooled effect size (*g* = 0.18 [0.04, 0.32]) across eight studies and high heterogeneity (*Q*[7] = 24.34, *p* =  < .01, *I*^*2*^ = 76.79%; see Fig. 3). Plurisexual groups scored higher on various measures of emotion awareness difficulties compared to monosexual minority groups with a small pooled effect size (*g* = 0.20 [0.06, 0.34]) across seven studies, with low heterogeneity (*Q*[6] = 5.11, *p* = .53, *I*^*2*^ = 0.00%). Transgender + groups also scored higher than cisgender groups on measures of emotion awareness difficulties, with a small pooled effect size (*g* = 0.25 [-0.08, 0.59]) across five studies with high heterogeneity (*Q*[4] = 20.76, *p* =  < .01, *I*^*2*^ = 75.01%). No studies compared the GSM combined groups with cisgender-heterosexual groups.

#### Acceptance of Emotion Responses

**Fig. 4:**
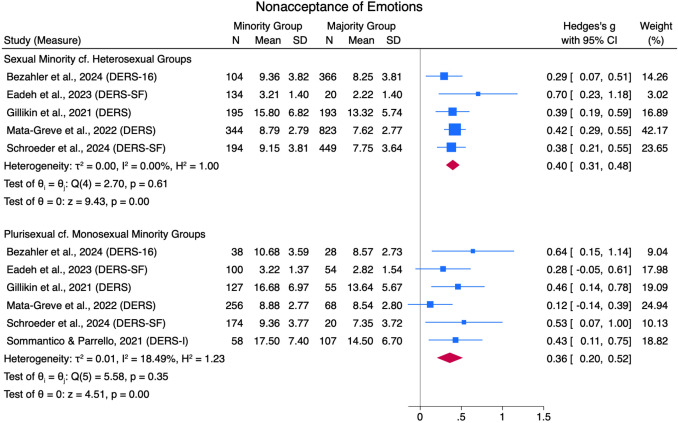
Nonacceptance of Emotions Forest Plot. *Note.* Random Effects REML Model. *N* = sample size. *M* = mean. *SD* = standard deviation. Emotion regulation measures: ADS = Affect Dysregulation Scale (Brown et al., 2012); BEIS = Brief Emotional Intelligence Scale (Davies et al., 2010); DERS = Difficulties in Emotion Regulation Scale (Kim L. Gratz & Roemer, 2004); DERS-SF = DERS Short Form (Kaufman et al., 2016); EES = Emotion Expression Scale for Children (Penza-Clyve & Zeman, 2002); TAS = The Toronto Alexithymia Scale (Bagby et al., 1994)

Sexual minorities scored higher than heterosexual groups on the DERS nonacceptance of emotions subscale, with a small effect size (*g* = 0.40 [0.31, 0.48]) across five studies with low heterogeneity (*Q*[4] = 2.70, *p* = .61, *I*^*2*^ = 0.00%; see Fig. 4). Plurisexual groups scored higher on the same nonacceptance of emotions subscale compared to monosexual minority groups in six studies with a small pooled effect size (*g* = 0.36 [0.20, 0.52]) and low heterogeneity (*Q*[5] = 5.58, *p* = .35, *I*^*2*^ = 18.49%). No studies compared this outcome for the remaining comparison groups.

#### Emotional Clarity

**Fig. 5:**
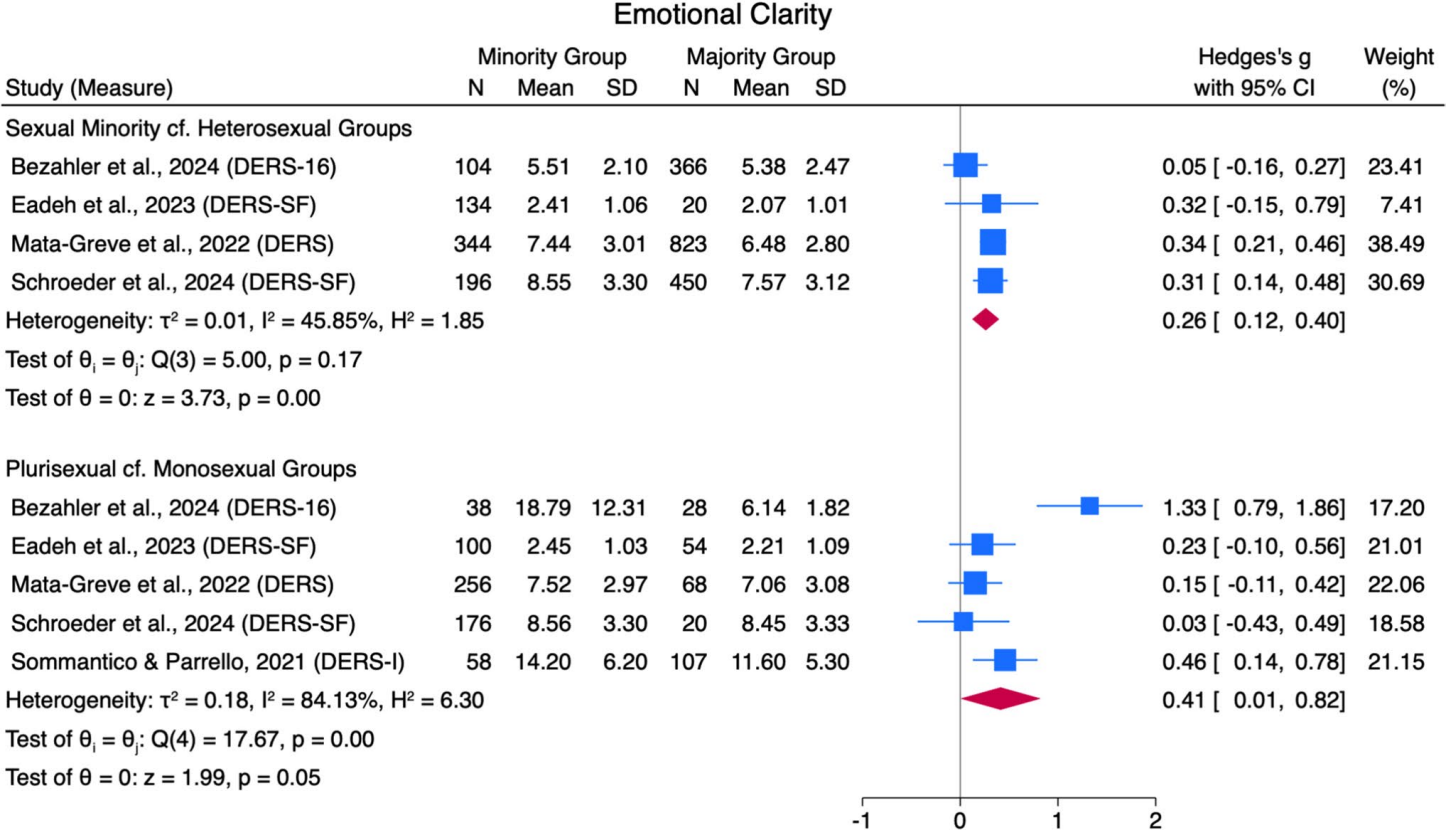
Emotional Clarity Forest Plot. *Note.* Random Effects REML Model. *N* = sample size. *M* = mean. *SD* = standard deviation. Emotion regulation measures: ADS = Affect Dysregulation Scale (Brown et al., 2012); BEIS = Brief Emotional Intelligence Scale (Davies et al., 2010); DERS = Difficulties in Emotion Regulation Scale (Gratz & Roemer, 2004); DERS-SF = DERS Short Form (Kaufman et al., 2016); EES = Emotion Expression Scale for Children (Penza-Clyve & Zeman, 2002); TAS = The Toronto Alexithymia Scale (Bagby et al., 1994)

Sexual minorities scored higher on the DERS difficulties with emotional clarity subscale compared to heterosexual people, with a small pooled effect size (*g* = 0.26 [0.12, 0.40]) across four studies and moderate heterogeneity (*Q*[3] = 5.00, *p* = .17, *I*^*2*^ = 45.85%; see Fig. 5). Plurisexual groups also scored higher than monosexual minority groups on emotional clarity difficulties, with a small pooled effect size (*g* = 0.41 [0.01, 0.82]) across five studies with high heterogeneity (*Q*[4] = 17.67, *p* =  < .01, *I*^*2*^ = 84.13%). No data for the other comparison groups for this outcome were found.

#### Engaging in Goal-Directed Behavior when Experiencing Emotions

**Fig. 6:**
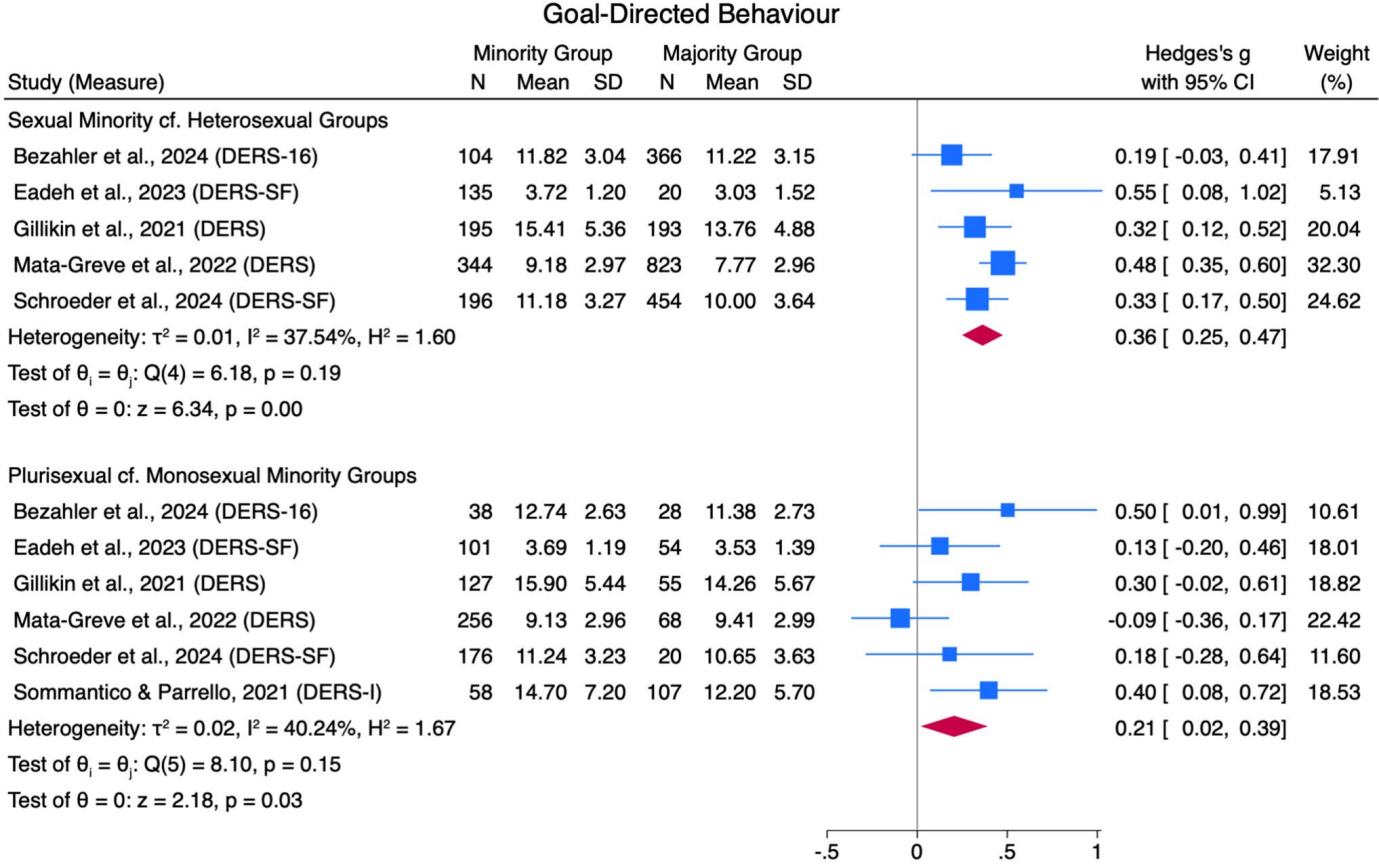
Goal-Directed Behavior Forest Plot. *Note.* Random Effects REML Model. *N* = sample size. *M* = mean. *SD* = standard deviation. Emotion regulation measures: ADS = Affect Dysregulation Scale (Brown et al., 2012); BEIS = Brief Emotional Intelligence Scale (Davies et al., 2010); DERS = Difficulties in Emotion Regulation Scale (Gratz & Roemer, 2004); DERS-SF = DERS Short Form (Kaufman et al., 2016); EES = Emotion Expression Scale for Children (Penza-Clyve & Zeman, 2002); TAS = The Toronto Alexithymia Scale (Bagby et al., 1994)

Five studies found that sexual minorities scored higher on the difficulties with engaging in goal-directed behaviors when experiencing emotions (DERS subscale) compared to heterosexual groups, with a small pooled effect size (*g* = 0.36 [0.25, 0.47]) and moderate heterogeneity (*Q*[4] = 6.18, *p* = .19, *I*^*2*^ = 37.54%; see Fig. 6). Six studies found that plurisexual groups scored higher on the same DERS subscale compared to monosexual minority groups, with a small pooled effect size (*g* = 0.21 [0.02, 0.39]) and moderate between-study heterogeneity (*Q*[5] = 8.10, *p* = .15, *I*^*2*^ = 40.24%). No studies compared this outcome for the remaining comparison groups.

#### Impulse Control when Experiencing Emotions

**Fig. 7:**
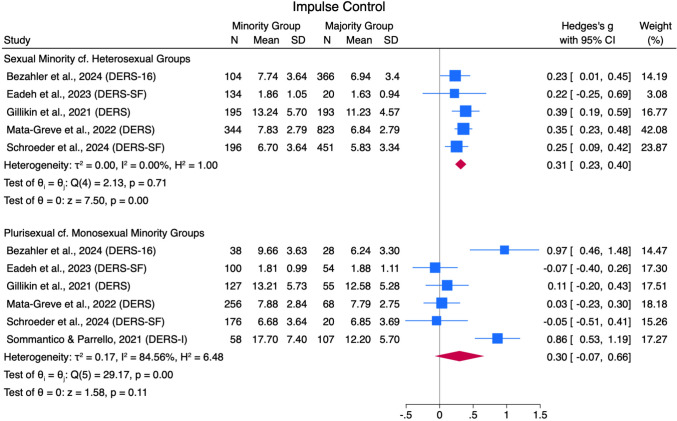
Impulse Control Forest Plot. *Note.* Random Effects REML Model. *N* = sample size. *M* = mean. *SD* = standard deviation. Emotion regulation measures: ADS = Affect Dysregulation Scale (Brown et al., 2012); BEIS = Brief Emotional Intelligence Scale (Davies et al., 2010); DERS = Difficulties in Emotion Regulation Scale (Gratz & Roemer, 2004); DERS-SF = DERS Short Form (Kaufman et al., 2016); EES = Emotion Expression Scale for Children (Penza-Clyve & Zeman, 2002); TAS = The Toronto Alexithymia Scale (Bagby et al., 1994)

Sexual minorities scored higher on impulse control difficulties (DERS subscale) compared to heterosexual people with a small pooled effect size (*g* = 0.31 [0.23, 0.40]) across five studies (low heterogeneity: *Q*[4] = 2.13, *p* = .71, *I*^*2*^ = 0.00%; see Fig. 7). Plurisexual people scored higher on the same DERS subscale compared to monosexual minority people, with a small pooled effect size (*g* = 0.30 [-0.07, 0.66]) across six studies and high heterogeneity (*Q*[5] = 29.17, *p* =  < .01, *I*^*2*^ = 84.56%). No identified studies compared this outcome for the remaining comparison groups.

#### Access to Emotion Regulation Strategies

**Fig. 8:**
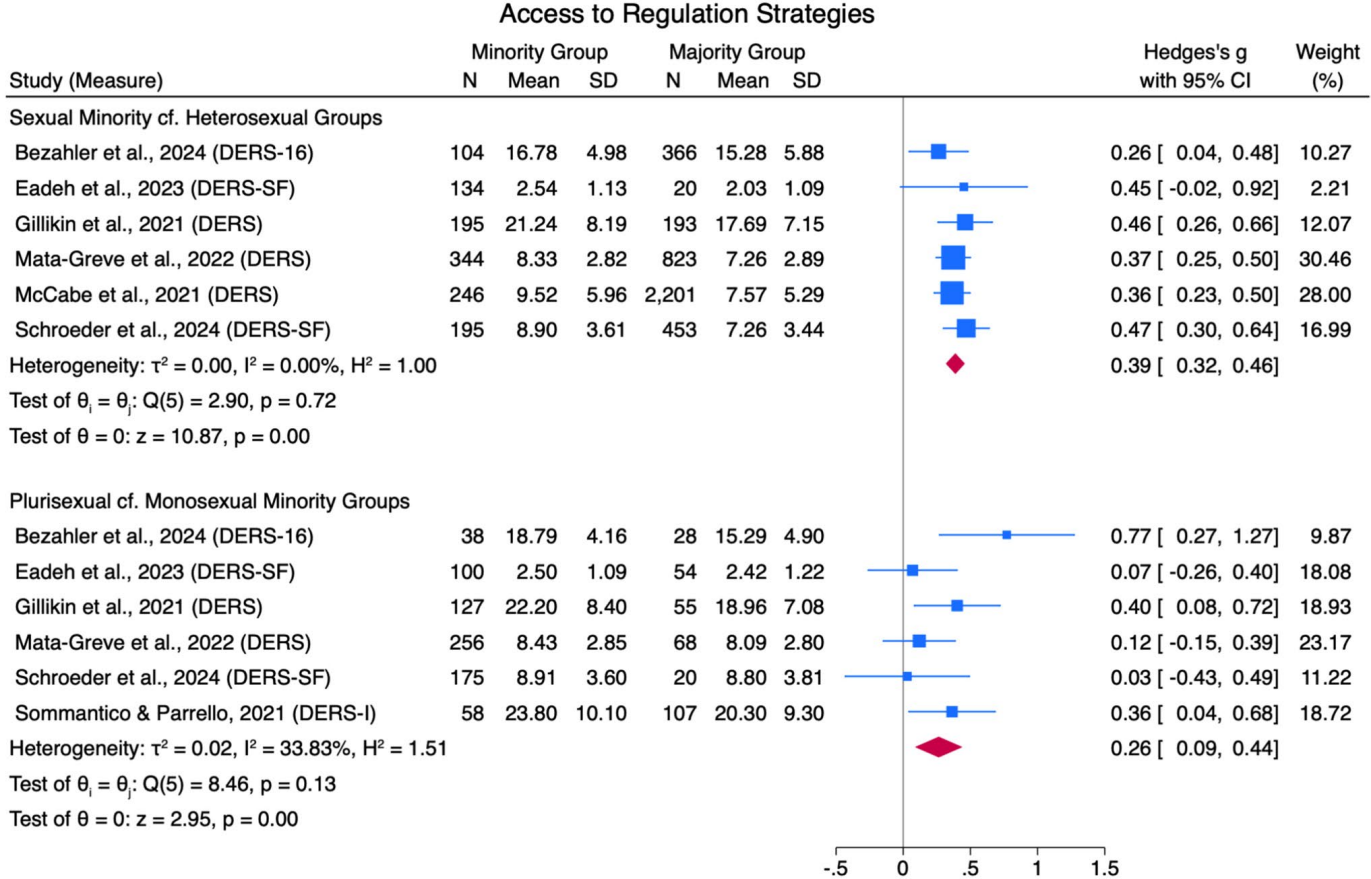
Emotion Regulation Strategies Access Forest Plot. *Note.* Random Effects REML Model. *N* = sample size. *M* = mean. *SD* = standard deviation. Emotion regulation measures: ADS = Affect Dysregulation Scale (Brown et al., 2012); BEIS = Brief Emotional Intelligence Scale (Davies et al., 2010); DERS = Difficulties in Emotion Regulation Scale (Gratz & Roemer, 2004); DERS-SF = DERS Short Form (Kaufman et al., 2016); EES = Emotion Expression Scale for Children (Penza-Clyve & Zeman, 2002); TAS = The Toronto Alexithymia Scale (Bagby et al., 1994)

Six studies found that sexual minorities scored higher on the difficulties with accessing emotion regulation strategies on the DERS subscale, with a small pooled effect size (*g* = 0.39 [0.32, 0.46]) and low heterogeneity (*Q*[5] = 2.90, *p* = .72, *I*^*2*^ = 0%; see Fig. 8). Six studies also reported that plurisexual groups scored higher on the same DERS subscale, with a small pooled effect size (*g* = 0.26 [0.09, 0.44]) and moderate heterogeneity (*Q*[5] = 8.46, *p* = .13, *I*^*2*^ = 33.83%). No further comparisons for this outcome were found.

#### Cognitive Reappraisal

**Fig. 9:**
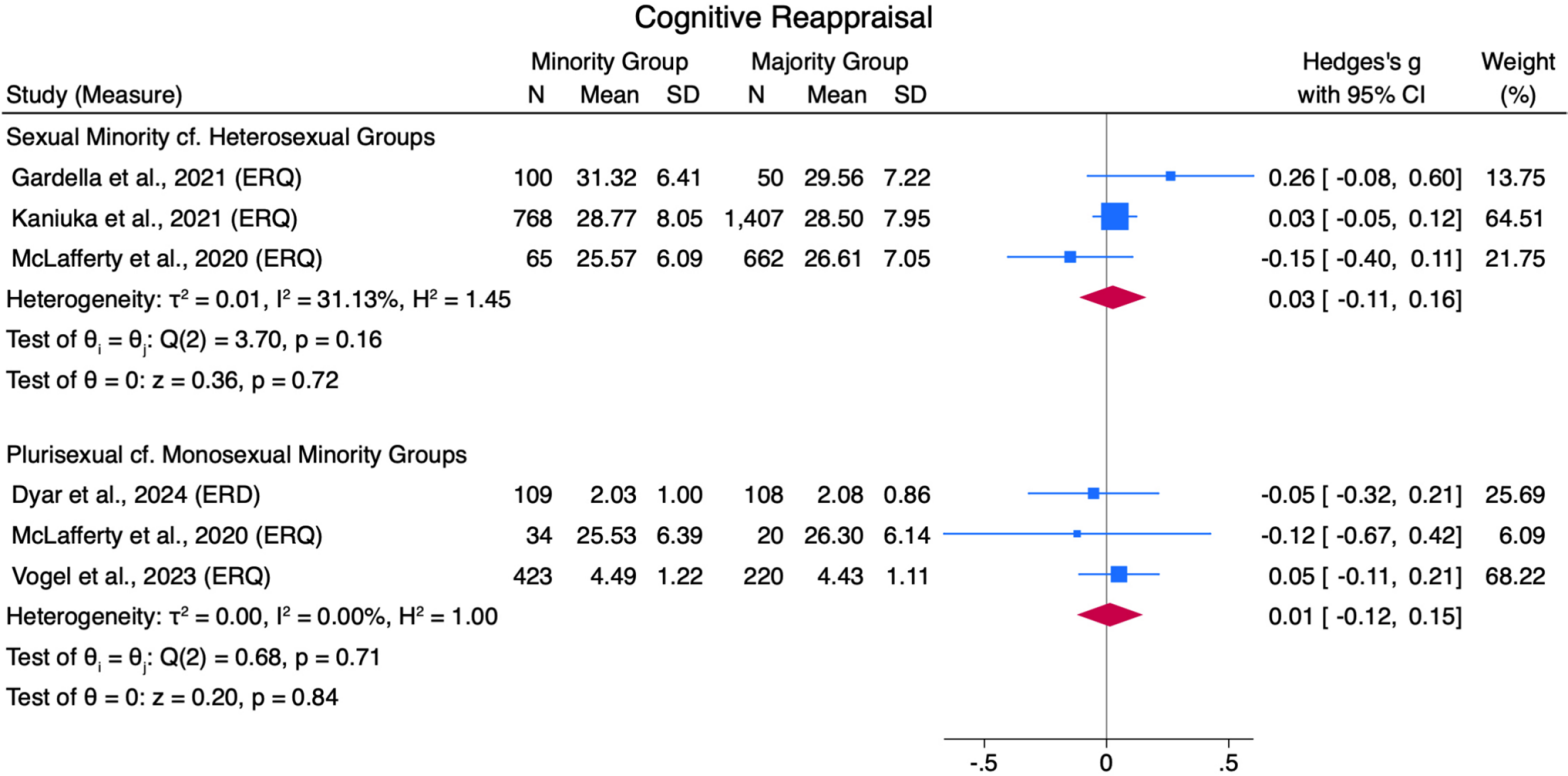
Cognitive Reappraisal Forest Plot. *Note.* Random Effects REML Model. *N* = sample size. *M* = mean. *SD* = standard deviation. Emotion regulation measures: ERQ = Emotion Regulation Questionnaire (Gross & John, 2003)

Negligible differences (*g* = 0.03 [− 0.11, 0.16] and *g* = 0.01 [− 0.12, 0.15], respectively) were found between sexual minorities and heterosexual groups, and plurisexual and monosexual minority groups, in their use of cognitive reappraisal to regulate emotions (ERQ), across three studies for each comparison, with moderate (*Q*[2] = 3.70, *p* = .16, *I*^*2*^ = 31.13%) to low (*Q*[2] = 0.68, *p* = .71, *I*^*2*^ = 0.00%) heterogeneity between studies, respectively (see Fig. 9).

#### Emotion Suppression

**Fig. 10:**
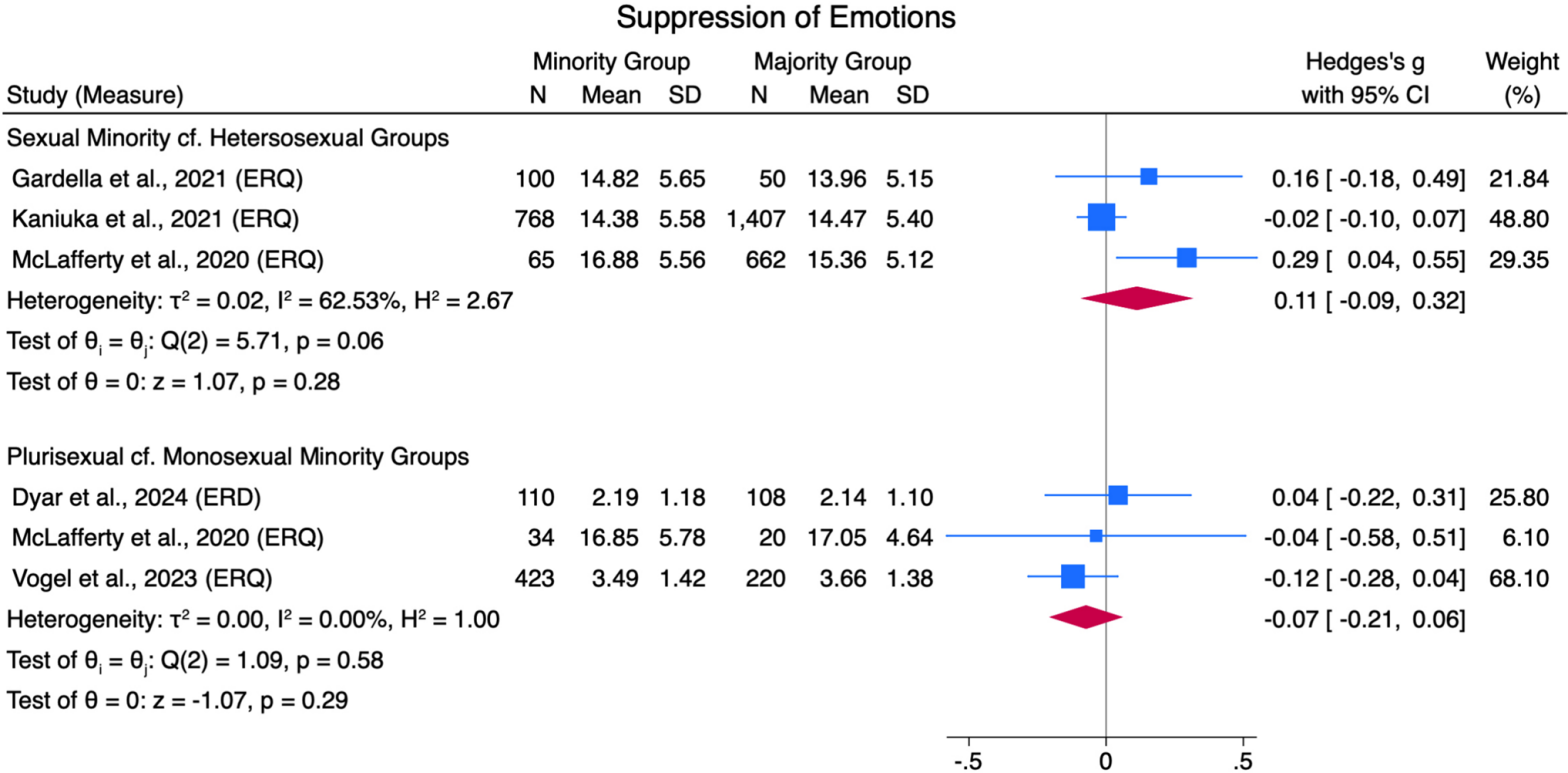
Suppression of Emotions Forest Plot. *Note.* Random Effects REML Model. *N* = sample size. *M* = mean. *SD* = standard deviation. Emotion regulation measures: ERQ = Emotion Regulation Questionnaire (Gross & John, 2003)

Negligible differences (*g* = 0.11 [− 0.09, .0.03] and *g* = − 0.07 [− 0.21, 0.06], respectively) between sexual minorities and heterosexual groups, and plurisexual and monosexual minority groups, were found in their use of emotion suppression (ERQ), across three studies for each comparison (see Fig. 10). The sexual minority and heterosexual group comparison had high heterogeneity (*Q*[2] = 5.71, *p* = .06, *I*^*2*^ = 62.53%), and the plurisexual and monosexual minority group comparison had low heterogeneity (*Q*[2] = 1.09, *p* = .58, *I*^*2*^ = 0.00%).

#### Rumination

**Fig. 11:**
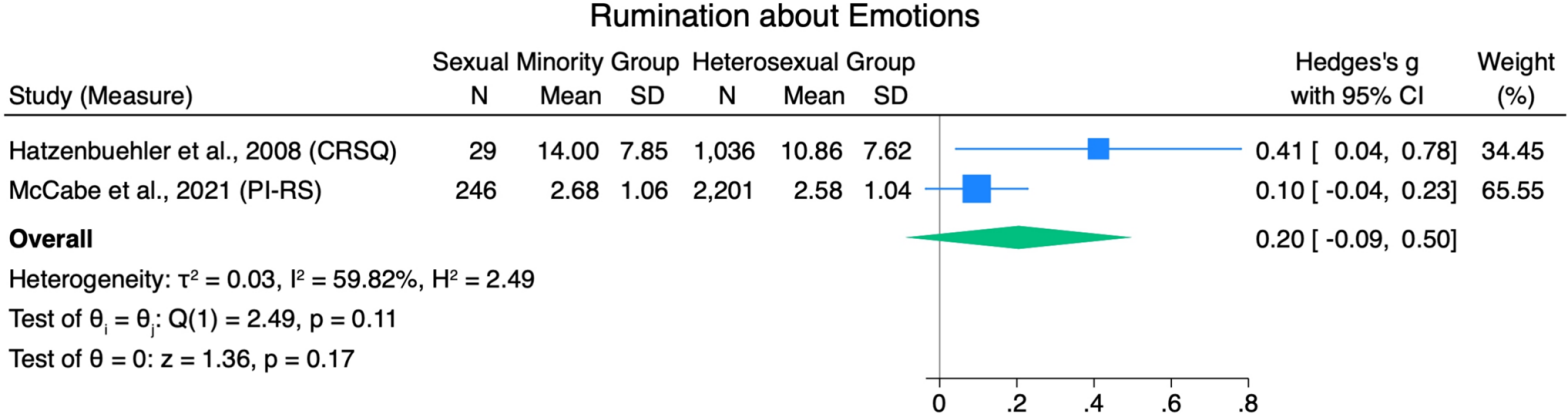
Rumination Forest Plot. *Note.* Random Effects REML Model. *N* = sample size. *M* = mean. *SD* = standard deviation. Emotion regulation measures: CRSQ = Children’s Response Styles Questionnaire (Abela et al., 2002); PI-R = Perfectionism Inventory: Subscale Rumination (Hill et al., 2004)

A pooled effect size from two studies suggested that sexual minorities scored higher on different measures of rumination associated with an attempt to regulate emotions compared to heterosexual groups, with a small pooled effect size (*g* = 0.20 [-0.09, 0.50]) and high heterogeneity (*Q*[1] = 2.49, *p* = .11, *I*^*2*^ = 59.82%; see Fig. 11).

### Sensitivity Analysis

A post-hoc sensitivity analysis conducting the meta-analysis with only the DERS or ERQ measure maintained largely consistent effect sizes and heterogeneity statistics (see Table 4). However, including consistent measures improved between-study heterogeneity for the sexual minorities and heterosexual group comparisons of global emotion regulation and the transgender + and cisgender comparison of emotion awareness. Conversely, analyzing consistent measures of global emotion regulation increased the between-study heterogeneity for the plurisexual and monosexual group comparison.

### Narrative Synthesis

Relevant findings from studies not included in the meta-analysis are summarized below.

#### Global Emotion Regulation

Two studies not included in the meta-analysis reported significantly higher global emotion dysregulation in sexual minority groups compared to heterosexual groups (Chang et al., 2020; Kapatais et al., 2023), aligning with the meta-analysis. Four studies found no significant differences in global emotion regulation scores between plurisexual groups and monosexual minority groups (English et al., 2018; Pachankis et al., 2015; Reitzel et al., 2017; Rogers et al., 2017).

Longitudinal findings indicated that those who consistently identified as a sexual minority over two years reported increasing emotion regulation difficulties relative to a heterosexual comparison group (Lopez et al., 2022). There were no differences between those who changed sexual identity over time and the consistently heterosexual group.

Ramos et al. (2022) found that cisgender-heterosexual boys scored lower on measures of emotion regulation difficulties than gender and sexuality diverse boys, followed by cisgender-heterosexual girls and then by gender and sexuality diverse girls, with the latter groups reporting progressively higher emotion regulation difficulties. The same study found that nonbinary young people scored significantly higher on emotion dysregulation compared to cisgender-heterosexual boys, but not compared to any other group.

Two studies reported that gender-diverse people scored higher on the DERS total score compared to cisgender-heterosexual people (Petruzzelli et al., 2022; Silveri et al., 2021a, 2021b), similar to findings in the meta-analysis. Another study found that transmen reported lower emotion dysregulation on the DERS than transwomen (Cao et al., 2023).

#### Specific Domains of Emotion Regulation

There were no significant differences in cognitive reappraisal and emotion suppression scores between masculine sexual minority women, feminine sexual minority women, or heterosexual women (Gardella et al., 2021), similar to findings in the meta-analysis. Another study found significantly higher scores for sexual minorities compared to heterosexual groups on emotion suppression (Teixeira et al., 2022), in contradiction to the meta-analysis, which found no pooled differences for this variable. No significant difference was found between these groups on cognitive reappraisal (Teixeira et al., 2022), in line with the meta-analysis findings.

One study found that plurisexual groups scored higher on rumination and a lack of emotional clarity compared to monosexual minority groups (Lattanner et al., 2022). Significant differences between plurisexual and monosexual minority groups in emotional clarity are congruent with the meta-analysis, whereas rumination did not have sufficient studies to be included in the meta-analysis for this comparison group.

Petruzzelli et al. (2022) also found that gender-diverse people scored higher on the nonacceptance of emotions DERS subscale and the difficulties engaging in goal-directed behavior, accessing strategies, and with emotional clarity subscales compared to cisgender-heterosexual people. No significant differences were found on the emotion awareness and impulse control subscales (Petruzzelli et al., 2022). Another study found no statistically significant difference in emotional suppression between trans men, cisgender men, or cisgender women (Warmuz-Stangierska et al., 2015).

#### Mediation Effects of Emotion Regulation

Three studies reported the mediating effect of emotion dysregulation in the relationship between GSM identity and mental health outcomes. In a serial mediation analysis, sexual minority status was associated with emotion dysregulation (Chang et al., 2020). Emotion dysregulation alone, and alongside social support, mediated the relationship between sexual minority status and suicide attempts (Chang et al., 2020). These findings were unchanged when controlling for gender and sampling method. Another mediation analysis found that emotion dysregulation mediated the relationship between GSM status and self-harm (Kapatais et al., 2023).

The final mediation analysis found that the nonacceptance of emotions, difficulties engaging in goal-directed behavior, impulse control, and access to regulation strategies (DERS subscales) mediated the relationship between sexual minority status and eating pathology while controlling for age and race (Gillikin et al., 2021). This was not statistically significant for the DERS awareness or clarity subscales.

## Discussion

This systematic review and meta-analysis examined differences in global and specific domains of emotion regulation between GSM and cisgender-heterosexual groups. This review also explored whether emotion dysregulation mediated any relationship between GSM identity and mental health outcomes. Generally, the findings supported the pattern that GSM individuals had increased difficulties with emotion regulation compared to cisgender-heterosexual individuals and a similar pattern for plurisexual groups (e.g., bisexual+ /pansexual) compared to monosexual minority groups (e.g., gay/lesbian).

### Emotion Regulation Differences Between Sexual Minority and Heterosexual Groups

Meta-analytic findings indicated that GSM groups overall and sexual minorities had higher global emotion dysregulation compared to their cisgender and heterosexual peers, with small pooled effect sizes. These findings were consistent with results from the narrative review and align with minority stress and psychological mediation theories (Cardona et al., 2022; Frost, 2011; Hatzenbuehler, 2009; Hendricks & Testa, 2012; Major & O’Brian, 2005; Meyer, 2003). These theories suggest that cumulative exposure to minority stressors and general stressors can compromise emotion regulation capacity, increasing the reliance on maladaptive coping behaviors.

Studies in the narrative synthesis also suggested that those who identify as sexual minorities consistently over time had more difficulties with emotion dysregulation compared to consistently heterosexual people and people who transitioned from a sexual minority to heterosexual. This may reflect the cumulative effects of ongoing minority stress on those who consistently identified as sexual minorities compared to those who consistently identified as or transitioned to a heterosexual identity (Lopez et al., 2022; Newcomb & Mustanski, 2010).

Sexual minorities also reported more difficulties in specific areas of emotion regulation, including nonacceptance of emotions; emotion clarity; goal-directed behavior and impulse control when experiencing emotions; access to regulation strategies; and rumination about emotions, with small pooled effect sizes. These domains are found to be associated with poorer mental health outcomes (Gratz et al., 2015, 2018; Gross, 2015a). Differences in cognitive reappraisal of emotions and emotion suppression between sexual minorities and heterosexual groups were negligible, suggesting these processes may be less central in explaining mental health disparities for this group. Most findings from the narrative review supported these patterns. However, one study reported higher emotion suppression in sexual minorities (Teixeira et al., 2022), contrasting with the meta-analysis results.

Differences in emotion awareness between sexual minorities and heterosexual groups were also negligible according to the meta-analysis. However, the pooled effect sizes were nearly at the threshold for a small difference between sexual minorities and heterosexual people. Difficulties with awareness of emotions were found to be significantly higher in some studies included in the meta-analysis, but not all. This was reflected in the high between-study heterogeneity in the meta-analysis for emotion awareness.

Overall, these findings suggest that specific domains of emotion regulation, such as clarity and acceptance, are more consistently impaired in sexual minorities and may therefore represent more promising intervention targets. However, as analyses including specific regulation domains were based on a limited number of studies, further research is needed to strengthen these conclusions.

### Emotion Regulation Differences Between Plurisexual and Monosexual Minority Groups

Meta-analytic findings indicated that plurisexual individuals had greater global and specific emotion regulation difficulties compared to monosexual minority individuals, with small pooled effect sizes. These results align with research highlighting the unique stressors faced by plurisexual people, including stigma and invalidation from both heterosexual and other sexual minority communities (Feinstein & Dyar, 2017; Ross et al., 2018a, 2018b; Wittgens et al., 2022). Such stressors may impair the development and use of adaptive regulation strategies. Differences in reported emotion dysregulation could also reflect variation in mental health disclosure and experiences of stigma between groups.

However, individual studies within the narrative synthesis did not find statistically significant differences in global emotion dysregulation (e.g., English et al., 2018; Pachankis et al., 2015) or nonacceptance of emotions (Petruzzelli et al., 2022) for this group comparison. This may be due to insufficient power to detect small effects in individual studies.

The meta-analysis also found negligible group differences when comparing emotion regulation strategies conceptualized within Gross’s (2003) model—namely cognitive reappraisal and suppression—which was supported by findings within the narrative synthesis. Therefore, the evidence suggests that emotion regulation domains most relevant to understanding health disparities in plurisexual populations may include emotion clarity, acceptance, access to strategies, and ability to engage in goal-directed behavior and managing impulsive urges in the context of intense emotions.

### Emotion Regulation Differences Between Transgender+and Cisgender Groups

The meta-analysis indicated that transgender+groups reported more difficulties with global emotion regulation than cisgender groups, with a small pooled effect size. This was supported by studies in the narrative synthesis. Minority stress theory highlights how chronic exposure to stigma-related and general stressors for transgender+populations can undermine the development of adaptive emotion regulation strategies (Frost, 2011; Hendricks & Testa, 2012). Given the high prevalence of sexual minority identities within transgender+populations, dual exposure to sexual- and gender-minority stressors may further compound emotion regulation difficulties (Reisner et al., 2023).

One study found that transgender men reported fewer difficulties with emotion regulation than transgender women (Cao et al., 2023). This aligns with evidence suggesting poorer mental health outcomes, increased self-harm, and greater exposure to childhood sexual abuse among female-assigned-sex transgender individuals compared to their male-assigned-sex counterparts (Rimes et al., 2019). However, more research is needed to clarify these within-group differences.

Transgender + participants also reported greater difficulties with emotion awareness compared to cisgender people in the meta-analysis, with a small effect size. However, this effect decreased below the threshold for a small effect in the sensitivity analysis, which only included studies using the DERS awareness subscale. This inconsistency was mirrored in the narrative synthesis, with one study finding no significant group difference. The limited number of studies and small transgender + sample sizes reduce the confidence in this finding, highlighting the need for more adequately powered research in this area.

Other emotion regulation domains could not be included in the meta-analysis due to insufficient studies. However, one narrative synthesis study found that transgender + individuals reported significantly higher difficulties with nonacceptance of emotions, emotion clarity, impulse control, goal-directed behavior engagement, and access to regulation strategies compared to cisgender individuals. Another study found no significant difference between transgender men, cisgender men, and cisgender women in emotion suppression (Warmuz-Stangierska et al., 2015). These findings suggest that while specific emotion regulation difficulties may be elevated in transgender+populations, more research is needed to establish consistent patterns—especially regarding underexplored domains such as rumination to regulate emotions, which may represent an important risk factor (Borders et al., 2014; Timmins et al., 2017).

In addition, differences may exist within transgender+and gender-diverse groups, such as between binary and nonbinary individuals (Rimes et al., 2019). Future research should prioritize within-group comparisons to better understand the diversity of emotion regulation across gender-diverse identities.

### The Mediating Effect of Emotion Regulation Between Gender and Sexual Minority Identity and Mental Health

Three studies investigated whether emotion regulation difficulties mediated the relationship between GSM identity and mental health outcomes. One study found that global emotion dysregulation mediated the relationship between sexual minority identity and suicide attempts (Chang et al., 2020), while another reported similar mediation effects for self-harm (Kapatais et al., 2023). A third study found that specific emotion regulation difficulties—namely nonacceptance of emotions, impulse control, goal-directed behavior, and access to strategies—mediated the relationship between sexual minority status and eating disorder symptoms (Gillikin et al., 2021). However, emotion awareness and clarity did not significantly mediate this relationship.

These findings support the potential role of emotion regulation as a key mechanism in understanding increased rates of adverse mental health outcomes for GSM groups. Given the elevated rates of self-harm, suicidal behaviors, and eating disorder symptoms in GSM populations (Liu et al., 2019; Parker & Harriger, 2020), identifying emotion regulation as a mediator offers important clinical and theoretical implications. Specifically, these findings are consistent with the Psychological Mediation Framework and other literature highlighting the mediating role of emotion regulation and its potential predictive role as a risk factor itself (Dyar, 2024; English et al., 2018; Fraser et al., 2018; Hatzenbuehler, 2009; Hatzenbuehler et al., 2008; Kapatais et al., 2023).

Nonetheless, further research is needed to determine whether these mediation effects extend to a wider range of mental health outcomes and to better understand potential differences across disaggregated GSM subgroups. This is important for informing more targeted, mechanism-based interventions aimed at reducing mental health disparities in GSM populations.

### Implications

The findings from this study suggest that emotion regulation is likely an important proximal risk factor contributing to mental health disparities in GSM populations. As such, emotion regulation represents a promising target for psychological interventions seeking to improve the health and well-being in this population. This aligns with existing evidence implicating emotion dysregulation in the development and maintenance of mental health difficulties (e.g., Kraiss et al., 2020) and its mediating role on the relationship between GSM stressors and mental health outcomes (e.g., Hatzenbuehler, 2009).

Strengthening emotion regulation capabilities may help GSM individuals to effectively navigate both general and minority stressors, fostering resilience in the face of cis-heterosexist environments. Importantly, this is not to de-emphasize the critical role of co-regulation and social support, which are also shown to buffer against poor mental health outcomes and serve as protective factors (Chang et al., 2020; Liu et al., 2019; Pachankis & Clark, 2025). Nor does it imply that the burden of managing systemic oppression should fall solely on the oppressed individuals. Individual-level interventions must be seen as complementary to broader sociopolitical efforts aimed at dismantling structural oppression. While such systemic change is often slow, it remains essential and has demonstrably broad benefits for both oppressed individuals and wider society (Badgett et al., 2019; Bauermeister, 2014; Camp, 2023; Camp et al., 2023; Raifman et al., 2017; Ramos et al., 2023).

Targeted interventions to bolster emotion regulation may help reduce disparities by decreasing difficulties in these areas to levels similar to cisgender-heterosexual peers. Culturally responsive therapeutic approaches for GSM individuals frequently emphasize building resilience, emotion coping capabilities, and community support (Austin et al., 2018; British Psychological Society, 2019; Lucassen et al., 2022; Nakamura et al., 2022; O'Shaughnessy & Speir, 2018; Pachankis et al., 2022; Skerven et al., 2021). Affirmative interventions often address emotion regulation components such as emotion awareness and acceptance, cognitive restructuring, strategies for modulating arousal, and problem solving (Austin et al., 2018; Lucassen et al., 2022; Pachankis et al., 2022; Skerven et al., 2021).

Evidence-based cognitive-behavioral interventions provide effective strategies to enhance capabilities—including increasing emotion awareness, cognitive reappraisal, and increasing goal-directed behavior—with growing evidence of efficacy in GSM populations (Aldao et al., 2014; Camp et al., 2024; Chang et al., 2023; Linehan, 1993; Papa et al., 2012). Particularly promising areas for interventions include increasing emotion clarity, increasing abilities to act in a goal-directed way, enhancing impulse control, accepting emotions, increasing access to adaptive regulation strategies, and reducing rumination about emotion-cueing stimuli. While cognitive reappraisal and emotion suppression showed negligible differences between GSM and cisgender-heterosexual populations, these processes nonetheless remain important for emotional well-being (Gross, 2015a, 2015b).

Further research is needed to investigate emotion regulation differences among understudied comparison groups, such as transgender + versus cisgender groups, or comparisons across sex assigned at birth. Research should also explore differences for varied minoritized groups, including GSM subgroups and other intersectionally diverse groups, given the differences found in this study and with other intersectionally diverse groups in other studies (e.g., GSM people of color; Chang et al., 2023; Cyrus, 2017; English et al., 2018; Feinstein & Dyar, 2017; Hoy-Ellis, 2023; Rimes et al., 2019; Ross et al., 2018a, 2018b; Wittgens et al., 2022. Additionally, future research should examine emotion regulation processes that were either underrepresented or absent in this review, such as problem solving as an emotion regulation strategy and rumination, given their potential importance (Borders et al., 2014; Gross, 2015a, 2015b; Timmins et al., 2017). More research is needed to investigate the role of emotion regulation as a predictor of a broader range of mental health outcomes in GSM populations.

### Limitations

The included studies had several limitations. Most relied on non-probability sampling methods, such as convenience sampling, which are unlikely to provide representative samples of the target population. Few studies reported a priori sample size justifications or considerations regarding statistical power, and data on non-responders were rarely provided. Despite this, most studies clearly defined their target population, utilized validated measures, and presented results clearly.

This review and meta-analysis also had several limitations. There was high heterogeneity between studies for some comparisons. While sensitivity analyses were conducted, heterogeneity persisted in several comparisons. Such methodological and population-level diversity is common in meta-analyses, and random effects models were used accordingly (Higgins et al., 2024). Additionally, some studies included in the meta-analysis were not originally designed to compare emotion regulation difficulties between groups but provided sufficient data either within their reporting or upon request. Therefore, group comparisons may not reflect optimally powered or specifically designed methodologies for this aim.

A further limitation relates to the aggregation of GSM identities. Many studies collapsed distinct identities into broader categories (e.g., sexual minority vs. gender minority), which may obscure important differences linked to other intersecting characteristics, such as sex, gender, or ethnicity/race—factors shown to influence minority stress, emotion regulation development, and mental health outcomes (Chang et al., 2023; Cyrus, 2017; English et al., 2018; Feinstein & Dyar, 2017; Hoy-Ellis, 2023; Rimes et al., 2019; Ross et al., 2018a, 2018b; Wittgens et al., 2022). This approach was necessary to enable quantitative synthesis, reflecting the limitations of the existing literature. However, future research should prioritize analyses of more disaggregated and intersectionally diverse subgroups.

Finally, very few studies investigated specific domains of emotion regulation. This limited the capacity of the review to explore differential patterns across emotion regulation domains and reveals a gap for future research.

### Conclusion

This systematic review and meta-analysis found that GSM groups, on average, experienced greater difficulties with global and specific domains of emotion regulation compared to cisgender and heterosexual groups. Plurisexual groups also had increased difficulties relative to monosexual minority groups. Although only a small number of studies examined emotion regulation as a mediating factor, initial evidence suggests it may help explain the increased mental health difficulties for GSM people compared to cisgender-heterosexual people. Therefore, these findings indicate the potential relevance of emotion regulation difficulties as both a mechanism underlying mental health disparities and a target for culturally responsive intervention. Further research is needed to confirm these associations and explore them across more diverse GSM subgroups and specific emotion regulation domains.

## Supplementary Information

Below is the link to the electronic supplementary material.

Supplementary file1 (DOCX 17 KB)

## Data Availability

The data availability will vary related to the original authors of the included studies. The descriptive data used for the meta-analyses are included in the forest plots.
