# Supplementary material for: Emotion Regulation Differences Between Gender and Sexuality Groups: A Systematic Review and Meta-Analysis
Source: Arch Sex Behav. 2025 Dec 16;54(10):3957–94. doi: 10.1007/s10508-025-03276-2 (PMC12753734; doi:10.1007/s10508-025-03276-2)
Supplement: Supplementary file 1 — Supplementary file1 (DOCX 17 KB) [file 10508_2025_3276_MOESM1_ESM.docx]

**Search Terms and Boolean Operators for OvidSP**

1 "emotion* *regulat*".ab,kf,kw,ti.

2 "emotion* control".ab,kf,kw,ti.

3 "affect* *regulat*".ab,kf,kw,ti.

4 "affect* control".ab,kf,kw,ti.

5 "self-regulat*".ab,kf,kw,ti.

6 "emotion* management".ab,kf,kw,ti.

7 "manag* emotion*".ab,kf,kw,ti.

8 "*regulat* emotion*".ab,kf,kw,ti.

9 "affect* management".ab,kf,kw,ti.

10 "manag* affect*".ab,kf,kw,ti.

11 "*regulat* affect*".ab,kf,kw,ti.

12 "distress *tolerance".ab,kf,kw,ti.

13 "tolerat* distress".ab,kf,kw,ti.

14 "emotion* modulat*".ab,kf,kw,ti.

15 "affect* modulat*".ab,kf,kw,ti.

16 "emotion* aware*".ab,kf,kw,ti.

17 "affect* aware*".ab,kf,kw,ti.

18 "emotion* intelligen*".ab,kf,kw,ti.

19 "affect* intelligen*".ab,kf,kw,ti.

20 "emotion* understand*".ab,kf,kw,ti.

21 "affect* understand*".ab,kf,kw,ti.

22 "emotion* clarity".ab,kf,kw,ti.

23 "affect* clarity".ab,kf,kw,ti.

24 "emotion* literacy".ab,kf,kw,ti.

25 "affect* literacy".ab,kf,kw,ti.

26 "emotion* knowledge".ab,kf,kw,ti.

27 "affect* knowledge".ab,kf,kw,ti.

28 "label* emotion*".ab,kf,kw,ti.

29 "label* affect*".ab,kf,kw,ti.

30 "alexithym*".ab,kf,kw,ti.

31 "emotion* recogn*".ab,kf,kw,ti.

32 "affect* recogn*".ab,kf,kw,ti.

33 "emotion* identi*".ab,kf,kw,ti.

34 "affect* identi*".ab,kf,kw,ti.

35 "emotion* accept*".ab,kf,kw,ti.

36 "affect* accept*".ab,kf,kw,ti.

37 "accept* emotion*".ab,kf,kw,ti.

38 "accept* affect*".ab,kf,kw,ti.

39 "reject emotion*".ab,kf,kw,ti.

40 "reject affect*".ab,kf,kw,ti.

41 "emotion* reject*".ab,kf,kw,ti.

42 "affect* reject*".ab,kf,kw,ti.

43 "emotion* tolerance".ab,kf,kw,ti.

44 "affect* tolerance".ab,kf,kw,ti.

45 "tolerat* emotion*".ab,kf,kw,ti.

46 "tolerat* affect*".ab,kf,kw,ti.

47 "goal-directed behav*".ab,kf,kw,ti.

48 "impulsiv*".ab,kf,kw,ti.

49 "planned behav*".ab,kf,kw,ti.

50 "behav* control".ab,kf,kw,ti.

51 "*regulat* strateg*".ab,kf,kw,ti.

52 "experience emotion*".ab,kf,kw,ti.

53 "experience affect*".ab,kf,kw,ti.

54 "LGB*".ab,kf,kw,ti.

55 "gay".ab,kf,kw,ti.

56 "lesbian".ab,kf,kw,ti.

57 "bisex*".ab,kf,kw,ti.

58 "queer".ab,kf,kw,ti.

59 "pansex*".ab,kf,kw,ti.

60 "homosex*".ab,kf,kw,ti.

61 "sexual minorit*".ab,kf,kw,ti.

62 "same sex attraction".ab,kf,kw,ti.

63 "sexual orientation".ab,kf,kw,ti.

64 "sexualit*".ab,kf,kw,ti.

65 "men who have sex with men".ab,kf,kw,ti.

66 "women who have sex with women".ab,kf,kw,ti.

67 "non-heterosex*".ab,kf,kw,ti.

68 "sexual preference*".ab,kf,kw,ti.

69 "sexual identit*".ab,kf,kw,ti.

70 "aromantic*".ab,kf,kw,ti.

71 "asexual*".ab,kf,kw,ti.

72 "demiromantic*".ab,kf,kw,ti.

73 "demisex*".ab,kf,kw,ti.

74 "romantic orientation".ab,kf,kw,ti.

75 "transgender*".ab,kf,kw,ti.

76 "transsex*".ab,kf,kw,ti.

77 "transmasculin*".ab,kf,kw,ti.

78 "transfeminin*".ab,kf,kw,ti.

79 "intersex*".ab,kf,kw,ti.

80 "gender dysphor*".ab,kf,kw,ti.

81 "gender identi*".ab,kf,kw,ti.

82 "gender nonconform*".ab,kf,kw,ti.

83 "gender minorit*".ab,kf,kw,ti.

84 "nonbinary".ab,kf,kw,ti.

85 "cisgender*".ab,kf,kw,ti.

86 "gender fluid*".ab,kf,kw,ti.

87 "gender varian*".ab,kf,kw,ti.

88 "gender divers*".ab,kf,kw,ti.

89 "gender express*".ab,kf,kw,ti.

90 "genderqueer".ab,kf,kw,ti.

91 "agender*".ab,kf,kw,ti.

92 "gender neutral*".ab,kf,kw,ti.

93 "third gender*".ab,kf,kw,ti.

94 "bigender*".ab,kf,kw,ti.

95 "neutrois".ab,kf,kw,ti. 4

96 "assigned male at birth".ab,kf,kw,ti.

97 "assigned female at birth".ab,kf,kw,ti.

98 "gender reassign*".ab,kf,kw,ti.

99 "gender incongruen*".ab,kf,kw,ti.

100 "heterosex* ".ab,kf,kw,ti.

101 1 or 2 or 3 or 4 or 5 or 6 or 7 or 8 or 9 or 10 or 11 or 12 or 13 or 14 or 15 or 16 or 17 or 18 or 19 or 20 or 21 or 22 or 23 or 24 or 25 or 26 or 27 or 28 or 29 or 30 or 31 or 32 or 33 or 34 or 35 or 36 or 37 or 38 or 39 or 40 or 41 or 42 or 43 or 44 or 45 or 46 or 47 or 48 or 49 or 50 or 51 or 52 or 53

102 54 or 55 or 56 or 57 or 58 or 59 or 60 or 61 or 62 or 63 or 64 or 65 or 66 or 67 or 68 or 69 or 70 or 71 or 72 or 73 or 74 or 75 or 76 or 77 or 78 or 79 or 80 or 81 or 82 or 83 or 84 or 85 or 86 or 87 or 88 or 89 or 90 or 91 or 92 or 93 or 94 or 95 or 96 or 97 or 98 or 99 or 10

103 101 and 102
